# Feeding the mind: preliminary insights into the effects of Anthocyanin-Rich Extract from black carrots on brain activity and gut microbiota in patients with cognitive impairments

**DOI:** 10.1038/s41598-025-29256-z

**Published:** 2025-11-27

**Authors:** Aynur Muduroglu-Kirmizibekmez, Alparslan Onder, Mustafa Yasir Ozdemir, Gamze Gurerk, Sevcan Aydin, Onder Yuksel Eryigit, Mehmet Oktar Guloglu, Ihsan Kara

**Affiliations:** 1 Department of Basic Science, Istanbul Nisantasi University, Istanbul, 34398 Turkey; 2 SANKARA Brain and Biotechnology Research Center, Istanbul, 34320 Turkey; 3 Department of Biomedical Technologies, Izmir Katip Celebi University, Izmir, 35620 Turkey; 4 Division of Biotechnology, Biology Department, Istanbul University, Istanbul, 34134 Turkey; 5 Vocational School of Health, Department of Anesthesiology and Reanimation, Istanbul Health and Technology University, Istanbul, 34275 Turkey; 6 Faculty of Medicine, Department of Histology and Embryology, Istanbul Aydin University, Istanbul, 34275 Turkey

**Keywords:** Anthocyanin, Cognitive Impairments, Gut-Brain Axis, EEG, Microbiota, Microbiome, Alzheimer's disease, Electroencephalography - EEG, Nutrition

## Abstract

Anthocyanins, known for their antioxidant and anti-inflammatory properties, have been associated with cognitive benefits, potentially mediated by gut-brain axis interactions. This study investigates the effects of a 12-week Anthocyanin-Rich Extract (ARE) intervention on brain activity and gut microbiota composition in older adults with neurocognitive impairments. In this study, 50 participants underwent electroencephalography (EEG) recordings and gut microbiota analyses before and after the intervention. EEG data were analyzed using connectivity and entropy metrics across multiple frequency bands. Gut microbiota composition was assessed via 16S rRNA sequencing to evaluate taxonomic shifts. Results revealed increased EEG connectivity, particularly in alpha, beta, and gamma frequency bands, suggesting improved neural communication and complexity following ARE consumption. Significant changes in nonlinear EEG metrics were observed, consistent with previous findings in the literature. Microbiota analysis indicated non-significant alterations in overall diversity but revealed increases in Alistipes, Streptococcus thermophilus, and Flavonifractor, alongside a decrease in Hungatella, potentially implicating SCFA metabolism and inflammatory regulation. These findings suggest ARE may enhance cognitive health by modulating neural activity and gut microbiota composition. While these results provide preliminary evidence of neuroprotective effects, further research with larger, disorder-specific cohorts and placebo-controlled designs is necessary to validate outcomes and explore gut-brain axis mechanisms in cognitive decline.

## Introduction

Neurocognitive disorders are increasingly prevalent among adults, posing a significant global health concern. Recent meta-analyses estimate that mild cognitive impairment (MCI)—a transitional stage between healthy aging and dementia—affects 15.56% of individuals aged 50 and older^1^. Another meta-analysis reported that within the same age group, the overall global prevalence of dementia was 6.97%, with Alzheimer’s disease (AD) accounting for 3.24% of cases^2^.

Given the rising incidence of cognitive decline, there is growing interest in dietary interventions that may help mitigate this risk. Among these, flavonoids—naturally occurring plant compounds with antioxidant and anti-inflammatory properties—have garnered attention for their potential neuroprotective effects. Anthocyanins, a subclass of flavonoids found in berries, grapes, and other pigmented fruits, have been linked to cognitive benefits in aging populations^3–9^.

Epidemiological research suggests that flavonoid-rich diets may offer protection against cognitive decline^10–24^. A widely recognized study from the Framingham Offspring Cohort, which tracked individuals over 19 years, found that participants consuming anthocyanin- and flavonoid-rich diets ($> 60\%$ of the cohort) had a significantly lower risk of developing dementia and Alzheimer’s disease compared to those with flavonoid-poor diets ($\le 15\%$ of the cohort)^25^.

Beyond their direct antioxidant and anti-inflammatory properties, flavonoids may also exert cognitive benefits through their interactions with the gut microbiome—a complex ecosystem of trillions of microorganisms that play a crucial role in overall health. In addition to supporting digestive function, the gut microbiome influences immune responses and neurological processes^26^. Recent research has highlighted the bidirectional communication between the gut microbiome and the central nervous system, known as the “gut-brain axis,” emphasizing its role in neurological function and cognitive health^26,27^. Dysbiosis, or an imbalance in gut microbiota, has been increasingly linked to neurodegenerative disorders, suggesting that gut health may be a critical factor in cognitive decline^28–31^.

The gut-brain axis plays a pivotal role in mediating the neuroprotective effects of anthocyanins, particularly in the context of neurodegenerative diseases. Zhong et al.^27^ demonstrated that anthocyanins modulate gut microbiota composition, thereby influencing gut-brain communication pathways and contributing to cognitive resilience. Similarly, Chen et al.^32^ found that gut microbiota-driven metabolic alterations in an Alzheimer’s disease mouse model significantly impacted brain function, further underscoring the gut’s role in neurodegeneration. Munir et al.^33^ suggested that targeting the gut-brain axis could restore the balance between beneficial and pathogenic microbial interactions, potentially mitigating neurodegenerative processes. Expanding on this, Sorboni et al.^34^ reviewed the therapeutic potential of microbiota modulation in neurological disorders, while Gallo et al.^35^ highlighted the association between MCI and gut microbiota, emphasizing microbiota-targeted interventions as a promising strategy for preserving cognitive health.

In this study, we examined the effects of a 12-week Anthocyanin-Rich Extract (ARE) intervention on older adults with varying degrees of cognitive impairment, all residing in stable living conditions.

ARE was extracted from black carrots (Daucus carota L. spp. sativus var. Atrorubens Alef.) using a modified method involving hot water extraction, acidification, and purification through a D-101 resin column, followed by ethanol elution and lyophilization to obtain a powdered form. The extract was then analyzed for its anthocyanin content using HPLC-DAD, total phenolic content via the Folin-Ciocalteu method, and antioxidant capacity using the CUPRAC assay, providing a comprehensive assessment of its bioactive properties.

To investigate the effects of ARE consumption on the gut-brain axis, we assessed changes in gut microbiota composition and neural activity. For this, we collected gut microbiome samples and resting-state electroencephalography (EEG) recordings from each participant before and after the intervention. Microbial composition was analyzed using 16S rRNA sequencing, a widely used method for characterizing gut microbiota diversity and taxonomic richness with high sensitivity^36^, allowing for a detailed evaluation of microbial changes pre- and post-intervention.

Aligned with our research objectives, EEG signal analysis incorporated a broad set of quantitative metrics to characterize brain activity and connectivity. Specifically, we employed the Imaginary Part of Coherence (iCOH) as the primary connectivity measure, along with Global Efficiency (GE), Local Efficiency (LE), and Transitivity (T) as key network measures. Additionally, to capture broader brain dynamics, we included Fractal Dimension (FD), Quadratic Sample Entropy (QSE), Quantile Graph (QG), and Visibility Graph (VG), as these metrics have well-documented relevance in EEG analysis. By leveraging this diverse analytical framework, we aimed to track changes in brain activity and connectivity over the 12-week ARE intervention.

While the cognitive benefits of anthocyanins have been widely recognized, the mechanisms underlying their effects, particularly through the gut-brain axis, remain underexplored. This study bridges this gap by investigating how anthocyanins influence various forms of cognitive impairment, emphasizing their potential role in microbiota-mediated neuroprotection. While prior research has primarily examined the link between dysbiosis and cognitive decline, our findings suggest that anthocyanins not only support cognitive health but may also serve as therapeutic agents. By reinforcing the importance of microbiota-targeted interventions, this study underscores the need for large-scale clinical trials and molecular-level investigations to elucidate these mechanisms further.

## Results

### Findings from quantitative EEG metrics

**Fig. 1:**
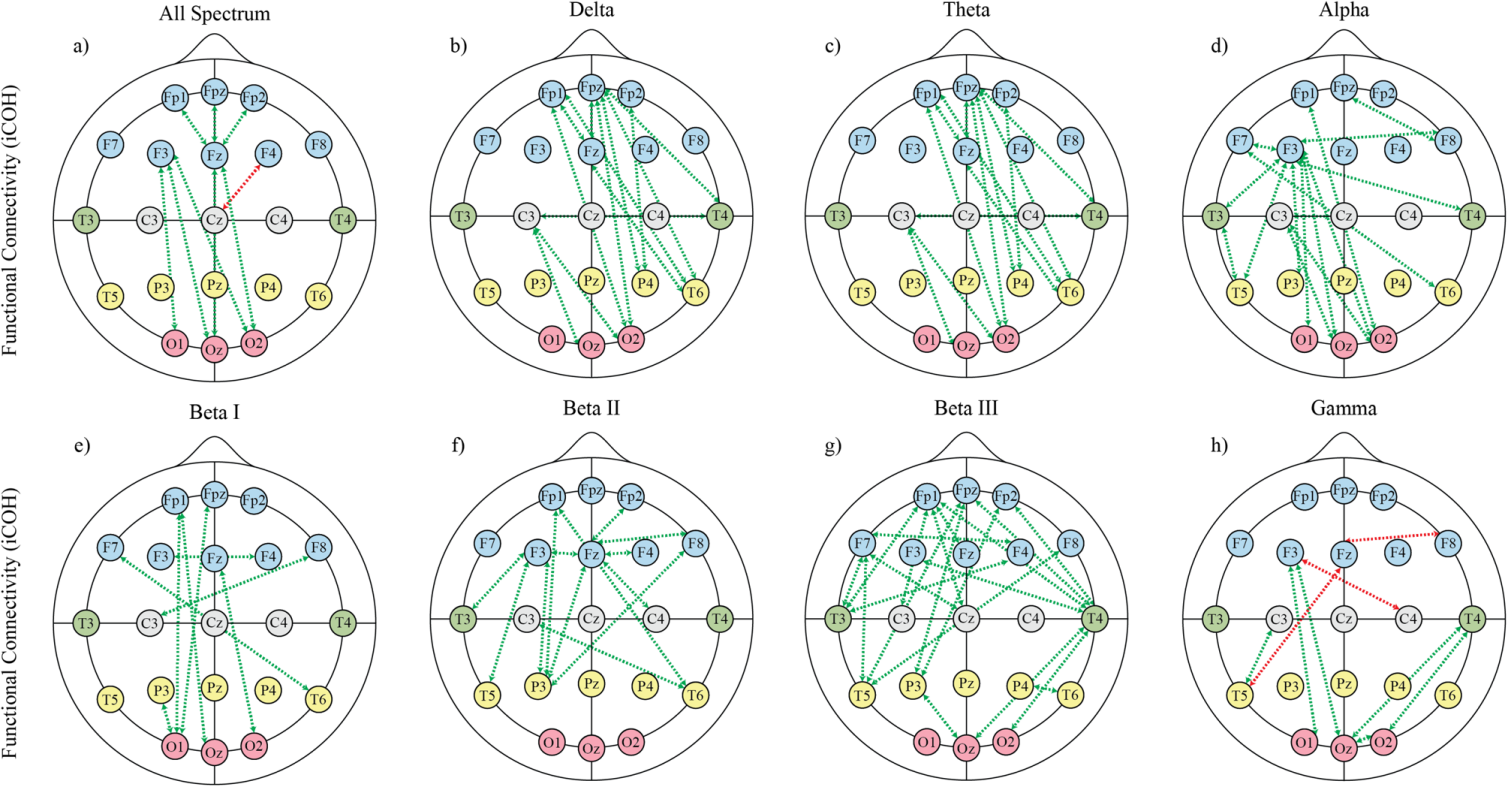
Functional Connectivity across different frequency bands computed with the iCOH. Panels (**A**–**H**) represent connectivity patterns in distinct EEG frequency bands: (**A)** All Spectrum (overall connectivity across all frequency bands), (**B)** Delta (1–3 Hz), (**C)** Theta (3–7 Hz), (**D)** Alpha (7–12 Hz), (**E)** Beta I (12–18 Hz), (**F)** Beta II (18–24 Hz), (**G)** Beta III (24–30 Hz), and (**H)** Gamma (30–45 Hz). Green lines indicate iCOH values that increased statistically significantly, while red lines indicate iCOH values that decreased statistically significantly. Each node represents an EEG electrode, color-coded by anatomical region (e.g., frontal, parietal, occipital, temporal).

**Fig. 2:**
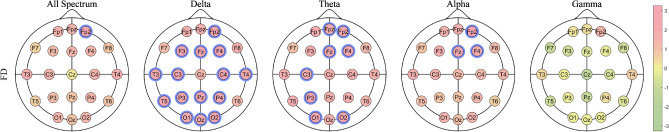
Changes in FD values before and after ARE consumption are visualized using z-score–based colormaps across selected EEG frequency bands: **All Spectrum** (1–45 Hz), **Delta** (1–3 Hz), **Theta** (3–7 Hz), **Alpha** (7–12 Hz), and **Gamma** (30–45 Hz). The Beta band was excluded due to a limited number of statistically significant findings. Statistically significant decreases in FD were predominantly observed in the Delta and Theta bands, particularly in frontal regions (e.g., Fp2, F4, Fz). In the All Spectrum analysis, Fp2 also exhibited a significant change. Electrodes with significant changes ($p < 0.05$, Wilcoxon signed-rank test) are highlighted in blue; however, none survived FDR correction at $q < 0.10$.

**Fig. 3:**
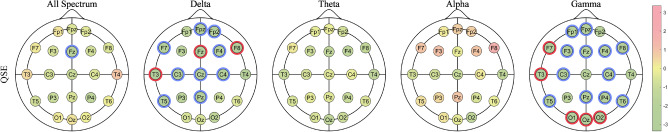
Changes in QSE values before and after ARE consumption are visualized using z-score–based colormaps across selected EEG frequency bands: **All Spectrum** (1–45 Hz), **Delta** (1–3 Hz), **Theta** (3–7 Hz), **Alpha** (7–12 Hz), and **Gamma** (30–45 Hz). The Beta band was excluded due to a limited number of statistically significant findings. Notable significant increases were observed in the central (Cz) and frontal (F8) regions in the Delta band, and in the occipital region (Oz) in the Gamma band. In the All Spectrum analysis, a significant increase was observed at Fz. Electrodes with statistically significant changes ($p < 0.05$, Wilcoxon signed-rank test) are highlighted in blue, while those surviving FDR correction at $q < 0.10$ are highlighted in red.

**Fig. 4:**
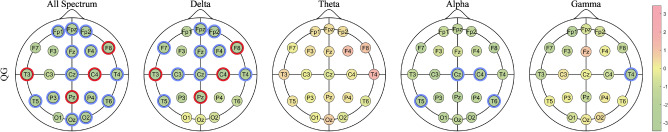
Changes in QG values before and after ARE consumption are visualized using z-score–based colormaps across selected EEG frequency bands: **All Spectrum** (1–45 Hz), **Delta** (1–3 Hz), **Theta** (3–7 Hz), **Alpha** (7–12 Hz), and **Gamma** (30–45 Hz). The Beta band was excluded due to a limited number of statistically significant findings. Most significant increases in QG were observed at frontal (F8), central (C4), and temporal (T3) electrodes in the Delta band. Widespread enhancements were also found in the All Spectrum, particularly at F8, C4, Pz, and Oz. Electrodes with statistically significant changes ($p < 0.05$, Wilcoxon signed-rank test) are highlighted in blue, while those surviving FDR correction at $q < 0.10$ are highlighted in red.

**Fig. 5:**
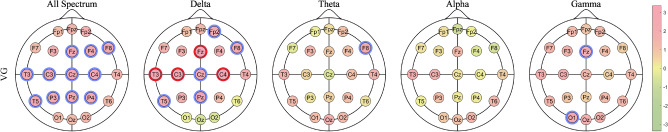
Changes in VG values before and after ARE consumption are visualized using z-score–based colormaps across selected EEG frequency bands: **All Spectrum** (1–45 Hz), **Delta** (1–3 Hz), **Theta** (3–7 Hz), **Alpha** (7–12 Hz), and **Gamma** (30–45 Hz). The Beta band was excluded due to a limited number of statistically significant findings. Significant decreases in VG were primarily observed at central (C4), frontal (Fz), and parietal (T5) electrodes, particularly in the Delta band and the All Spectrum. In the Gamma band, a significant decrease was noted at Fz and O1. Electrodes with statistically significant changes ($p < 0.05$, Wilcoxon signed-rank test) are highlighted in blue, while those surviving FDR correction at $q < 0.10$ are highlighted in red.

We employed Wilcoxon’s signed-rank test on results for EEG metrics iCOH, GE, LE, T, FD, QSE, QG, and VG across both conditions. This was to determine whether the metrics had statistically significant differences before and after consumption of ARE. This process was repeated for the results of each frequency band. The significant changes obtained are given in Figs. 1, 2, 3, 4, and 5.

Figure 1 presents connectivity patterns derived from the iCOH across eight EEG frequency bands. Statistically significant increases and decreases in functional connectivity between electrode pairs are visualized with green and red lines, respectively, allowing for topological comparisons across bands. Each electrode is color-coded based on anatomical region to aid interpretation of regional connectivity dynamics.

Figures 2 through 5 illustrate the spatial distributions on scalp maps of changes in FD, QSE, QG, and VG before and after ARE consumption. These metrics are visualized using z-score–based topographic colormaps across selected frequency bands (Delta, Theta, Alpha, Gamma, and the full spectrum). In the colormaps, increases and decreases relative to baseline are represented in red and green, respectively, according to the direction of the z-score. Electrodes showing statistically significant differences ($p < 0.05$, Wilcoxon signed-rank test) are highlighted in blue, and those that remain significant after Benjamini–Hochberg FDR (False Discovery Rate) correction, $q < 0.10$) are shown in red, indicating the possible anatomical regions that may have been affected following ARE intake.

#### Results for delta frequency band (1–3 Hz)

The analysis of the delta frequency band indicated a non-significant increase in GE ($\hbox {z} = -1.37$, $p = 0.168$) and T ($\hbox {z} = -1.14$, $p = 0.251$). Out of 21 channels, LE showed an increase in 20, with channel T5 demonstrating a significant increase ($\hbox {z} = -2.39$, $p = 0.016$). Conversely, channel P3 showed a non-significant decrease ($\hbox {z} = 0.43$, $p = 0.067$). In the iCOH analysis, out of 210 channel connectivity combinations, 47 decreased non-significantly, while 163 increased, with 15 significant increases. Notable significant increases were observed in the connectivity between several brain regions as shown in Fig. 1B.

FD showed a significant decrease across all electrodes except Fp1, Fpz, F7, F8, Cz, and T6 as shown in Fig. 2. The most significant difference for FD in the delta band was observed at the F4 electrode ($\hbox {z} = 2.53$, $p = 0.001$). Nevertheless, none of these effects survived FDR correction.

QSE significantly increased in the central region (most notably at CZ; $\hbox {z} = -2.65$, $p = 0.008$) and the frontal region (most notably at F8; $\hbox {z} = -2.85$, $p = 0.004$). In addition to these dominant alterations, notable changes can also be observed in the prefrontal, right and left frontotemporal, and left temporal regions, as illustrated in Fig. 3. After FDR correction, only electrodes Fz, F8, and T3 (respectively, $q = 0.075, 0.083, 0.075$) remained significant.

The locations of the significant differences in QG were similar to those observed in QSE (Fig. 4). Specifically, an increase was noted in the frontal region (most notably at F8; $\hbox {z} = -2.85$, $p = 0.004$) and the central region (most notably at C4; $\hbox {z} = -2.80$, $p = 0.005$). The most significant increase was observed at the T3 ($\hbox {z} = -3.05$, $p = 0,002$). F8, T3, C4 and Pz (respectively, $q = 0.094, 0.094, 0.094, 0.094$) electrodes remained significant after FDR correction.

VG showed a significant decrease in the central area, with the most significant decrease observed on the C4 electrode ($\hbox {z} = 3.35$, $p = 0.001$). As illustrated in Fig. 5, significant decreases were also found at the Fp2, F8, Fz, T3, T5, and Pz electrodes. All changes in VG were negative, except for those observed in the occipital region. After FDR correction, only electrodes Fz, T3, C3, and C4 (respectively, $q = 0.077, 0.074, 0.074, 0.074$) remained significant.

#### Results for theta frequency band (3–7 Hz)

The analysis of the theta frequency band showed a non-significant increase in both GE ($\hbox {z} = -1.47$, $p = 0.142$) and T ($\hbox {z} = -1.47$, $p = 0.139$). All channels exhibited an increase in LE, with channels Fpz ($\hbox {z} = -2.25$, $p = 0.024$) and C3 ($\hbox {z} = -2.23$, $p = 0.026$) demonstrating significant. In the iCOH analysis, mirroring the delta band results, 47 connectivity combinations decreased non-significantly, while 163 increased, with 15 significant increases. Notable significant ones were observed between the areas, as depicted in Fig. 1C.

FD showed a significant decrease in the prefrontal region (most notably at Fp2; $\hbox {z} = 2.55$, $p = 0.011$) and the occipital region (most notably at O1; $\hbox {z} = 2.43$, $p = 0.015$), as well as at the Fz ($\hbox {z} = 2.59$, $p = 0.009$), F4 ($\hbox {z} = 2.46$, $p = 0.014$), C3 ($\hbox {z} = 1.97$, $p = 0.048$), and P3 ($\hbox {z} = 2.50$, $p = 0.012$) electrodes, as illustrated in Fig. 2. As illustrated in Fig. 5, the only significant change in VG was observed at the F8 electrode ($\hbox {z} = 2.07$, $p = 0.038$). While no significant differences were found in QSE or QG in the theta frequency band, both exhibited an increasing trend, whereas VG tended to decrease.

#### Results for alpha frequency band (7–12 Hz)

In the alpha frequency band, both GE and T exhibited non-significant increases ($\hbox {z} = -0.84$, $p = 0.403$ and $\hbox {z} = -1.27$, $p = 0.201$, respectively). LE increased in 20 of the 21 channels, with channel F3 being the only significant one ($\hbox {z} = -2.11$, $p = 0.035$). Conversely, channel F4 showed a non-significant decrease ($\hbox {z} = 0.526$, $p = 0.599$). The iCOH analysis revealed 53 non-significant decreases in connectivity combinations and 157 increases, with 16 of these increases being significant. Notably, significant increases were observed between various areas, as illustrated in Fig. 1D.

FD showed a decrease across all channels, with significant decreases observed at the Fp2 ($\hbox {z} = 2.45$, $p = 0.014$), Fz ($\hbox {z} = 2.23$, $p = 0.025$), and F4 ($\hbox {z} = 2.07$, $p = 0.038$) electrodes, as illustrated in Fig. 2. In contrast, QG showed an overall increase across all channels, with significant increases observed at the Cz ($\hbox {z} = -2.48$, $p = 0.013$), C4 ($\hbox {z} = -2.10$, $p = 0.035$), T5 ($\hbox {z} = -2.35$, $p = 0.019$), and T6 ($\hbox {z} = -2.07$, $p = 0.038$) electrodes, as shown in Fig. 4.

No significant differences were observed in QSE or VG. In QSE, the largest difference was at the F8 electrode ($\hbox {z} = 1.65$, $p = 0.010$), and the direction of the changes varied across regions. These changes can be followed through the color variations depicted in Fig. 3. QSE decreased in the frontal region (excluding Fz) while increasing in other regions (excluding C4, P3, Pz, and O1). In VG, the largest difference was observed at the T3 electrode ($\hbox {z} = 1.84$, $p = 0.065$), with a decrease across most electrodes, except at Fpz, Fp2, F4, F8, C4, and Pz.

#### Results for beta I frequency band (12–18 Hz)

In the beta I frequency band, both GE and T exhibited non-significant changes ($\hbox {z} = -0.76$, $p = 0.447$ and $\hbox {z} = -1.75$, $p = 0.451$, respectively). LE showed increases in 20 out of 21 channels, with a non-significant reduction observed in channel Pz ($\hbox {z} = 0.526$, $p = 0.599$). The iCOH analysis identified 77 non-significant reductions in connectivity combinations and 133 increases, 8 of which were significant. Significant increases were particularly noted between specific areas, as shown in Fig. 1E.

No significant differences were observed in FD, QSE, QG, or VG at the beta I frequency band. The channels with the lowest p-values were Fp2 ($\hbox {z} = 1.64$, $p = 0.099$), Pz ($\hbox {z} = 1.69$, $p = 0.090$), T5 ($\hbox {z} = 1.42$, $p = 0.154$), and F8 ($\hbox {z} = 1.65$, $p = 0.098$) for FD, QSE, QG, and VG, respectively. When examining the z-values of FD and QG, it was seen that these two features exhibit different trends between the low-frequency and high-frequency bands. This difference starts to be seen in the Beta1 frequency band. The decreasing trend in FD turns into an increasing trend in T5, Pz, and central regions and spreads to all other regions as the frequency increases. The increasing trend seen in VG starts to decrease except for T6 and O2 electrodes, but unlike FD, this trend narrows in regions with increasing frequency.

#### Results for beta II frequency band (18–24 Hz)

In the beta II frequency band, both GE and T exhibited non-significant changes ($\hbox {z} = -0.72$, $p = 0.473$, and $\hbox {z} = -1.00$, $p = 0.321$, respectively). LE showed increases across 20 channels, with a significant increase at channel F3 ($\hbox {z} = -2.38$, $p = 0.017$), while channel Pz showed a non-significant reduction ($\hbox {z} = 0.265$, $p = 0.791$). The iCOH analysis revealed 43 non-significant reductions in connectivity combinations and 167 increases, 13 of which were statistically significant. Significant increases in connectivity were observed between specific regions, as shown in Fig. 1F.

No significant differences were found in FD, QSE, QG, or VG, except for the Fp2 electrode in FD ($\hbox {z} = 2.1$, $p = 0.035$). The channels with the lowest p-values were O1 ($\hbox {z} = -1.65$, $p = 0.099$) for QSE, Fz ($\hbox {z} = -1.38$, $p = 0.166$) for QG, and O1 ($\hbox {z} = 1.57$, $p = 0.117$) for VG. In FD, in addition to the changes observed in beta I, the direction of change at the Fpz, F7, F3, and F8 electrodes shifted from decreases to increases.

#### Results for beta III frequency band (24–30 Hz)

In the beta III frequency band, both GE and T showed significant increases ($\hbox {z} = -2.18$, $p = 0.029$, and $\hbox {z} = -2.22$, $p = 0.028$, respectively). LE exhibited increases across all 21 channels, with significant increases at Fp1, Fpz, Fp2, F3, Fz, F8, T3, and C3. The iCOH analysis revealed 32 non-significant reductions in connectivity combinations and 178 increases, 22 of which were statistically significant. Significant increases in connectivity were observed between various regions, as shown in Fig. 1G.

No significant differences were observed in FD, QSE, QG, or VG at the beta III frequency band. The channels with the lowest p-values were Fp2 ($\hbox {z} = 1.81$, $p = 0.070$) for FD, F4 ($\hbox {z} = -1.62$, $p = 0.106$) for QSE, C3 ($\hbox {z} = -1.91$, $p = 0.055$) for QG, and Fp2 ($\hbox {z} = 1.88$, $p = 0.060$) for VG. In FD, in addition to locations where the changes were observed in beta I and beta II, increases were observed at the Fz, P3, and T6 electrodes.

#### Results for gamma frequency band (30–45 Hz)

In the gamma frequency band, both GE and T showed insignificant increases ($\hbox {z} = -0.01$, $p = 0.991$, and $\hbox {z} = -0.04$, $p = 0.973$, respectively). LE increased in 13 of the 21 channels, though these changes were insignificant. Conversely, insignificant decreases in LE were observed in channels F7, Fz, F4, F8, T3, Cz, C4, and Pz. The iCOH analysis revealed 100 decreased connectivity combinations, three of which were significant. Additionally, 110 increases in connectivity were identified, with six being significant. These significant increases occurred between specific areas, as shown in Fig. 1H.

FD showed no significant differences in the gamma frequency band. In contrast, QSE exhibited significant increases across most electrodes, except for Fp2, Cz, T8, and P3, as shown in Fig. 3. The occipital region, however, demonstrated particularly significant increases (most notably at Oz; $\hbox {z} = -3.25$, $p = 0.001$). Following FDR correction, significant increases persisted at F7, T3, O1, Oz, and O2 (respectively, $q = 0.083, 0.083, 0.075, 0.075, 0.085$). QG showed only a single significant difference, an increase at the T8 electrode (Fig. 4), whereas VG exhibited decreases across all electrodes, with significant decreases observed at the Fz and O1 electrodes (Fig. 5).

#### Results for entire spectrum (1–45 Hz)

Our analysis of the full frequency spectrum (1–45 Hz) revealed several key observations. Although the GE demonstrated a slight increase, it was not statistically significant ($\hbox {z} = -0.6$, $p = 0.549$). Similarly, T showed a small, insignificant increase ($\hbox {z} = -0.55$, $p = 0.589$). LE exhibited increases in 18 out of 21 channels, none of which were statistically significant, while decreases were observed in three channels (F4, Cz, and Pz), also without significance. The iCOH analysis identified 81 instances of decreased connectivity combinations, with one being significant, alongside 129 increased connectivity values, 8 of which were significant. These changes were noted between specific regions, as depicted in Fig. 1A.

In FD, a significant difference was found only at the Fp2 ($\hbox {z} = 2.60$, $p = 0.009$) electrode (Fig. 2), whereas QSE displayed a significant difference at the Fz ($\hbox {z} = -1.98$, $p = 0.047$) electrode (Fig. 3). QG exhibited significant increases across all electrodes (most notably frontal electrode F8; $\hbox {z} = -3.44$, $p = 0.001$; most notably central electrode C4; $\hbox {z} = -2.81$, $p = 0.005$; most notably parietal electrode Pz; $\hbox {z} = -2.87$, $p = 0.004$; most notably occipital electrode Oz; $\hbox {z} = -2.08$, $p = 0.038$) except F7, F3, C3, and O1, as illustrated in Figure 4. Additionally, after FDR correction, electrodes F8, T3, C4, and Pz (respectively, $q = 0.094, 0.094, 0.094, 0.094$) remained significant. VG showed significant decreases in the central (most notably at C4; $\hbox {z} = 2.55$, $p = 0.011$), right frontal (most notably at Fz; $\hbox {z} = 2.82$, $p = 0.005$), and parietal (most notably at T5; $\hbox {z} = 2.50$, $p = 0.012$) regions, as depicted in Fig. 5. The FD and VG values decreased, while the QSE and QG values increased across all electrodes for the entire spectrum.

### Gut microbiota analysis findings

**Fig. 6:**
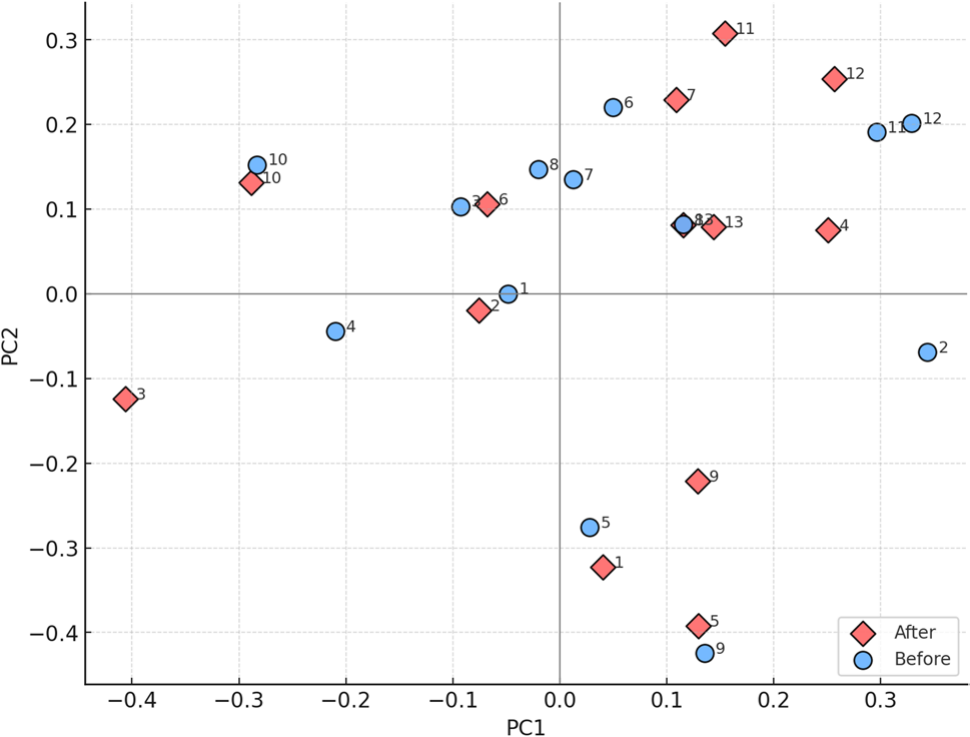
PCoA analysis of samples with Bray–Curtis analysis (beta diversity), shown along the first two principal coordinates (PC1 and PC2). Each subject is represented by paired measurements before (skyblue circles) and after (salmon diamonds) the intervention, labeled with subject IDs (1–13). The ordination illustrates within-subject trajectories and overall differences in beta diversity between pre- and post-intervention states.

The gut microbiota analysis revealed no statistically significant changes in alpha diversity measures, including the Chao1 index, Shannon index, and Simpson index. The results of the pairwise Kruskal–Wallis tests are presented in Table 1. Similarly, beta diversity metrics showed no significant differences, with the Bray–Curtis PCoA (Fig. 6) and Jaccard indices. Detailed results of the pairwise PERMANOVA tests are provided in Table 2.

**Table 1:** Pairwise Kruskal–Wallis test results for alpha diversity indices.

Alpha diversity measures	H	p-value	q-value
Chao1 index	0.039	0.852	0.852
Shannon index	0.015	0.901	0.901
Simpson index	0.044	0.833	0.833

**Table 2:** Pairwise PERMANOVA results for beta diversity metrics.

Beta diversity metrics	pseudo-*F*	p-value	q-value
Bray-Curtis PCoA index	0.934	0.542	0.542
Jaccard index	0.015	0.884	0.884

Although the detailed analysis revealed no statistically significant changes, it identified four dominant phyla across all groups: *Actinomycetota*, *Bacillota*, *Bacteroidota*, and *Pseudomonadota*. Among these, *Actinomycetota* and *Bacillota* decreased by 37% and 9%, while *Bacteroidota* and *Pseudomonadota* increased by 18% and 70%, respectively. *Actinomycetota* exhibited a 37% decrease, primarily driven by a 43% reduction in the *Coriobacteriia* class.

Within the *Bacillota* phylum, the *Bacilli* class showed a 25% decrease, influenced by a 38% reduction in *Enterococcus faecium*. Additionally, the *Lactobacillaceae* family exhibited a 46% decrease. In contrast, the *Streptococcus* genus increased by 6%, with *Streptococcus thermophilus* showing a 248% increase.

The *Clostridia* class demonstrated an overall decrease of 7.3%, driven by a 7.2% reduction in the *Eubacteriales* order. Decreases were observed across several genera, including a 72% decrease in *Roseburia*, 20% in *Coprococcus*, and 18% in *Lachnoclostridium*. Within the genus *Lacrimispora*, a 70% reduction was observed, largely driven by a 72% decrease in the species *Lacrimispora saccharolytica*. The genus *Hungatella* showed a 33% decrease, mirrored by its species *Hungatella hathewayi* (33%). Similarly, *Enterocloster* exhibited a 41% decrease, with *Enterocloster bolteae* reducing by 53%. The genus *Anaerostipes* decreased by 24%, primarily due to a 57% reduction in the species *Anaerostipes hadrus*. The genus *Blautia* experienced a 25% decrease, with reductions in *Blautia parvula* (52%) and *Blautia pseudococcoides* (81%). Additionally, *Faecalibacterium* showed a 54% decrease, while its species *Faecalicatena sp. Marseille-Q4148* decreased by 16%.

Conversely, certain genera displayed increases. The *Flavonifractor* genus increased by 133%, driven by the species *Flavonifractor plautii* (129%). The *Vescimonas* genus increased by 66%, with *Vescimonas coprocola* contributing a 125% increase. Additionally, *Clostridiales bacterium* and *Clostridiales bacterium CCNA10* species showed increases of 48% and 76%, respectively. For the *Erysipelotrichia* class, a 198% increase was observed. This was primarily driven by the *Erysipelotrichaceae* family, which exhibited a 131% increase, suggesting it had the major potential contribution to this change.

**Fig. 7:**
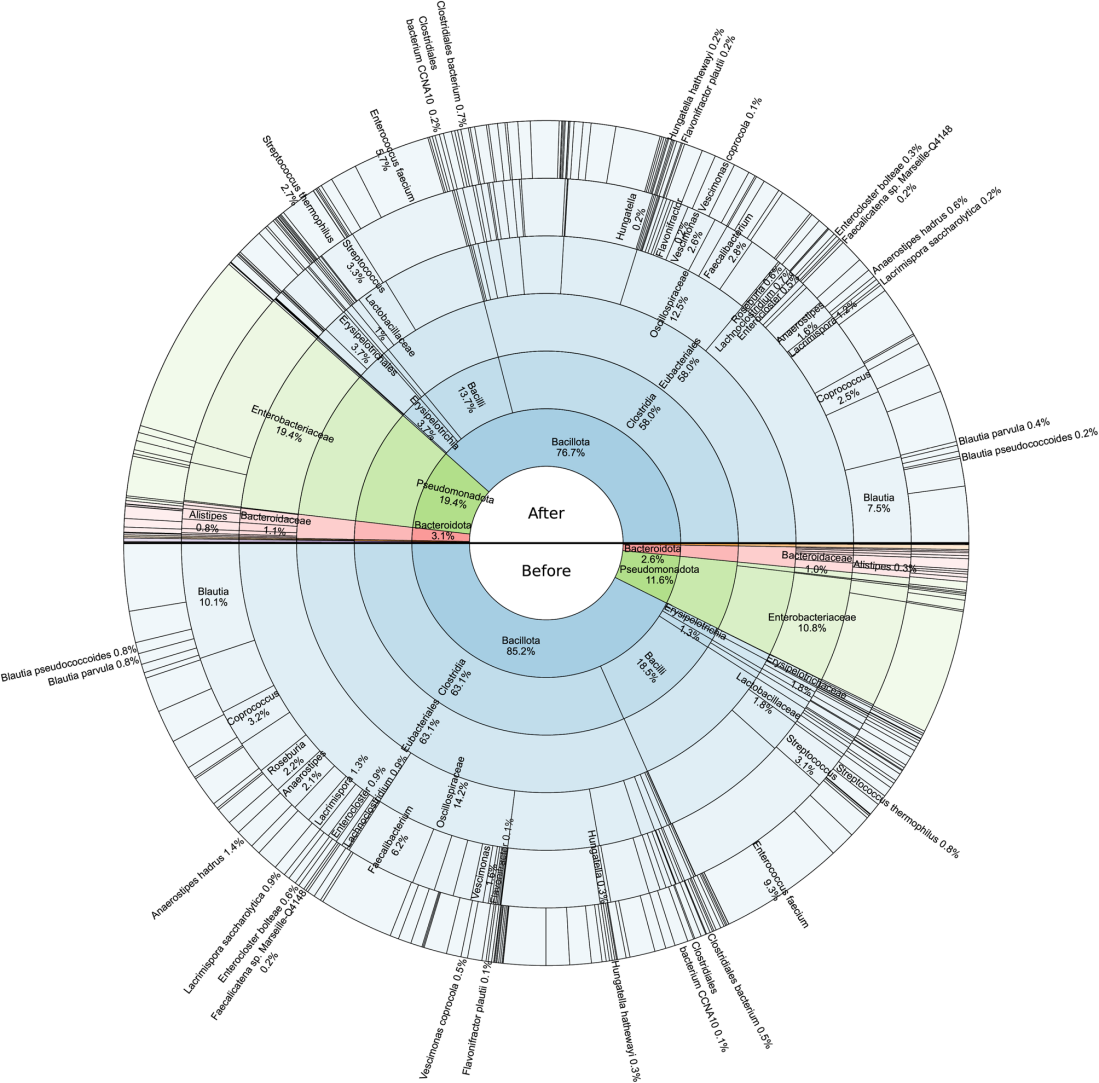
Differential taxonomic composition before and after ARE consumption. The circular hierarchical chart represents taxonomic changes in the gut microbiota composition at various taxonomic levels, comparing “Before” and “After” ARE consumption. The inner circle represents the broader taxonomic levels (e.g., phylum), while the outer layers represent finer taxonomic classifications (e.g., genus and species). The proportions of different taxa at the same level are displayed as percentages.

For the *Bacteroidota* phylum, an overall increase of 18% was observed, strongly influenced by a 226% increase in the *Alistipes* genus. In contrast, the *Bacteroidaceae* family showed a 39% decrease. Finally, for the *Pseudomonadota* phylum, a 70% increase was noted, primarily driven by an 80% increase in the *Enterobacteriaceae* family. Whole changes before and after ARE consumption are shown in Fig. 7.

### Correlation analysis results

Correlation analyses between changes in nonlinear EEG metrics (FD, QSE, QG, VG) and relative shifts in gut microbial taxa did not yield statistically significant associations after FDR correction of Kendall’s tau correlations. However, feature–taxon pairs with uncorrected $p < 0.001$ in Kendall’s test were further evaluated using Spearman’s rank correlation. These exploratory results are presented in the Supplementary Materials. Notably, a correlation was identified between FD and the class *Coriobacteriia*, as shown in the supplementary correlation table. Although the overall abundance of *Coriobacteriia* decreased after ARE intake, this taxon retained its correlations with EEG metrics.

## Discussion

Our study demonstrated that 12 weeks of ARE supplementation led to increased EEG connectivity across alpha, beta, and gamma frequency bands, as well as notable changes in nonlinear EEG metrics. Gut microbiota analysis revealed no significant shifts in overall diversity but showed increases in Alistipes, Streptococcus thermophilus, and Flavonifractor, along with a decrease in Hungatella. These findings suggest that anthocyanins may exert neuroprotective effects by enhancing neural communication and modulating gut microbial composition. In the context of existing literature, previous studies in the field of functional connectivity, particularly within lower frequency bands such as delta and theta, have reported.

In the field of functional connectivity, researchers have identified a wide range of outcomes, particularly in the lower frequency bands, such as delta and theta^37–40^. Notably, Musaeus et al. have documented a decline in iCOH, correlating with the progression of Alzheimer’s disease^41^. However, the increased iCOH values observed in our study suggest a potentially beneficial impact of anthocyanin molecules on individuals with varying degrees of cognitive impairment.

In the alpha frequency band, contrary to previous findings of reduced coherence in cognitive impairments^42–48^, our study reveals a notable increase in functional connectivity, as indicated by elevated iCOH values, among elderly individuals following a 12-week ARE supplementation regimen. This finding highlights the potential therapeutic effects of anthocyanins in supporting brain function in this population.

Similarly, while multiple studies have consistently reported a decrease in functional connectivity within the beta frequency band^38,43,49–51^ as cognitive impairments progress, our study observed increased functional connectivity in the beta I (12–18 Hz), beta II (18–24 Hz), and beta III (24–30 Hz) frequency bands among elderly individuals with cognitive impairments following a three-month ARE supplementation regimen.

Although the existing literature does not provide specific findings on gamma band connectivity in relation to cognitive impairment, one study noted that adults exhibit lower beta and gamma connectivity than children, which has been associated with brain aging^52^. The increased gamma band connectivity observed in our study following ARE supplementation may serve as evidence of its positive effects.

Previous studies have suggested a decline in GE within the delta band with the development of cognitive impairments^39,53^. Therefore, it is plausible that the consumption of ARE might lead to a modest yet noteworthy increase in GE within this frequency range. Our study found non-significant increases in GE in the delta band after ARE usage, aligning with observations of potential beneficial effects on cognitive function. In the theta frequency band, while some investigations have reported higher GE values among healthy individuals compared to those with cognitive impairments^54–57^, others have noted a decrease in functional connectivity among Alzheimer’s disease patients^50,58^. Given the subtle uptick in GE values among individuals with cognitive impairments in our study, it’s plausible to speculate that ARE consumption could positively influence their cognitive functions. Similar to delta, our results showed non-significant increases in GE in the theta band.

For the alpha frequency band, while some investigations have echoed previous research by highlighting a decline in GE values among elderly individuals with cognitive impairments^54,59^, our study yields a strikingly different result. We observed that the consumption of ARE facilitated a discernible increase in GE values. In the beta frequency band, some research has highlighted a decline in GE with the onset of cognitive impairments^53^. However, the GE values in our study’s cohort increased insignificantly in the beta I and beta II frequency bands. In the beta III (24–30 Hz) frequency band, our study’s participants exhibited significant increases in GE values. For the gamma frequency band, while some studies have reported a reduction in GE among elderly individuals with cognitive impairments^60^, our study indicates increased GE values, suggesting potential benefits of ARE.

In the beta III (24–30 Hz) frequency band, LE demonstrated a significant increase across eight channels, indicating that ARE consumption might have a positive impact on local connectivity within this frequency range. For the gamma frequency band, some studies have indicated higher LE among elderly individuals with cognitive impairments^54^, while others suggest a reduction^60^. Our study reveals increased LE values, suggesting potential benefits from ARE usage.

Our study found non-significant increases in T in the delta band after ARE usage, aligning with observations of potential beneficial effects on cognitive function. Similar trends were observed in the theta band, where results indicated non-significant increases in T, suggesting the potential benefits of anthocyanin on visual activation. Studies suggest a decrease in fronto-parietal connectivity in the theta band correlates with visual activation and cognitive decline^61^. In the beta III (24–30 Hz) frequency band, our study’s participants exhibited significant increases in T values, further supporting the potential positive impact of ARE on cognitive function.

For the entire spectrum (1–45 Hz), the overall increase in connectivity metrics observed may serve as evidence of the positive effects of ARE.

The EEG traces show a noticeable decrease in signal complexity as the progression of diseases accompanied by cognitive impairment such as dementia and Alzheimer’s disease. According to some studies, this decrease in complexity is related to the loss of synaptic connections, leading to decreased functional connectivity and a slowing of the brain’s oscillatory activity. The study of Zappasodi et al. examining neuronal impairment after stroke supports this view^62^. Neuronal and synapse loss after stroke is a known condition and it has been determined that FD is lower in individuals who have had a stroke regardless of location in the study. In a study evaluating the performance of different FD algorithms, the Katz FD values were lower in dementia patients compared to normal subjects^63^. A previous study observed that patients with dementia have lower values than normals for different FD algorithms^64^. Also some studies show that FD decreases with AD^65,66^. These studies found a significant difference in FD values between the healthy and the AD subjects. However, a conflicting result was observed in a study by Vicchietti et al.^67^. When we examined the FD values based on frequency, the changes observed in the delta, theta, and alpha bands were consistent with the findings of Vicchietti et al. but did not align with the expected results from other studies. Especially in delta, the direction of changes was consistent with the findings of Vicchietti et al.^67^, but not with those reported in the study by Ahmadlou et al.^65^, which used the same database. The increment trend in FD, starting from the beta I band and continuing to rise into the gamma band, is consistent with those studies. It can be interpreted that while complexity increases in high frequencies, it decreases in low frequencies such as theta and delta. The increase in complexity is considered a more active physiological marker, and it is observed that magnitudes representing complexity, such as FD, decrease in cases of cognitive impairment. In this context, we expected an increase in FD using ARE; however, this was not observed in the entire spectrum signal. However, considering the literature on increased slow wave activity (delta, theta) and decreased high wave activity (alpha, beta, gamma) in Alzheimer’s patients^68^, it can be said that the use of ARE, considering in frequency bands, has an effect opposite to the course of the disease.

With a similar approach, the ability of QSE to measure regularity in the signal is associated with signal complexity in AD patients^69,70^. The review^70^, which examined 40 different studies on various entropy calculation methods, indicates that entropy decreases with the severity of cognitive impairment. According to Simons et al.^69^, lower QSE was observed in AD patients except for the F3 and T3 electrodes. The QSE values we obtained were parallel to these results. In particular, the QSE value increased in the delta and gamma bands, where significant differences were observed. In the study conducted by Vicchietti et al.^67^, a significant difference was observed in the F7 electrode, which showed the highest difference, before and after the use of ARE. The F7 electrode was highlighted in another study^71^ due to its proximity to the hippocampal region. This region, which is related to memory and learning, is considered to be the first region to begin to deteriorate in AD patients. For this reason, it is thought that the changes occurring in this region are important. QSE is a robust entropy measure, therefore, assuming that the effects of noise-induced distortion are low, it can be said that high changes, especially in the gamma band, indicate an increase in energy level. Considering that high-frequency components are associated with the cortex region, changes in high-level cognitive activities are expected.

In this study, the parameter QG represents the average jump length for the network created by the QG transformation. According to the study conducted by Pineda et al.^71^, this feature is higher in AD patients than in the healthy control group. In addition, while the values obtained with eyes open and closed are more discriminatory in healthy groups, this difference is quite small in AD patients. This situation is related to the weakness of the alpha band, and the low alpha waves indicate that the effective global synchronization of the default cortical network is impaired^72^.

While outcomes of both studies of Pineda et al.^71^ and Vicchietti et al.^67^ demonstrated the efficiency of the QG as a distinctive feature for the healthy control group and AD patients; Pineda et al.^71^ underlined the difference was more significant in the delta frequency band in addition to entire spectrum (1–45 Hz). These findings are in line with our results. However, QG value increased after ARE consumption, we expected that QG would decrease like the healthy control group in Pineda’s work. Although current studies are insufficient to interpret changes in QG as an indicator of cognitive impairment, these changes may still be associated with its progression.

The graph index complexity (GIC) parameter was used for VG, the last nonlinear method we used to transform EEG time series into a network structure. In the study conducted by Ahmadlou et al.^73^, a significant decrease was observed in the alpha band in AD patients with eyes closed in F3, P3, and F8 electrodes, and a significant increase was observed in theta and delta bands in P4, Fz and P3, O2, T6 electrodes, respectively. Although not exactly overlapping, the following similar results were obtained; VG value decreases in theta and delta bands, and the majority of the increased VG values are in the alpha band. In the study conducted by Vicchietti et al.^67^, VG values were found to be lower in AD patients. Although the values we found do not agree with the study in this respect, it is noteworthy that focusing the significant differences on the delta band. The lower these values are, the more they are associated with the decrease in complexity. However, relating each nonlinear feature to complexity term and then to functional connectivity constitutes a problematic approach. For this reason, it would be a more accurate approach to interpret these nonlinear features by considering what they represent.

FD is largely associated with repeating structures. The VG is sensitive to rapid and sudden changes in signals, besides is associated with fractal features. In addition, QSE is a term related to complexity and is evaluated similarly to FD. However, QSE and QG are more prone to average values. The GIC parameter used for the VG value is more sensitive to peak values in time series signals. When these are considered, the decreases in FD and VG and the increases in QSE and QG in the delta band, where the most significant changes are seen; suggest that the waves belonging to the delta frequency are more stable waves that do not contain sudden changes, do not have multiple growing or shrinking peaks, and do not shift too much in the range of the delta frequency. A similar situation is also seen in the theta band, although it does not have significant changes. It is stated in the literature that high activity in slow waves may be related to cholinergic deficit in AD patients, and symptomatic treatments such as cholinesterase inhibitors (ChEIs) have been tried in this regard. In this context, anthocyanin which we think causes a more regular slow wave course, may be an alternative^74^. Studies on cholinergic transmission also support this view. In contrast, when compared to the literature, we see that FD, QSE, QG, and VG values, especially in the delta and theta bands, are mostly parallel to AD disease^75^. However, as we go to higher frequencies, especially in the gamma band, an activity in the opposite trend is observed. This situation gives rise to the idea that after the use of ARE in patients with cognitive impairments, the course of the disease continues but some changes occur in terms of cognition and symptomatic.

Our EEG findings indicate that ARE supplementation positively influences both functional connectivity and signal complexity across multiple frequency bands. While lower frequency bands (delta and theta) exhibited modest, non-significant changes, higher frequencies (alpha, beta, and gamma) showed notable increases in connectivity (iCOH, GE, LE, T) and complexity-related metrics (QSE, QG). These results suggest that ARE may enhance neural network efficiency and counteract some of the connectivity disruptions associated with cognitive impairment. Although further research is needed to confirm these effects, the overall findings support the potential cognitive benefits of ARE supplementation.

An increasing number of studies have demonstrated significant alterations in the structure and composition of the gut microbiota in individuals with cognitive impairment^30^. This study aimed to investigate the effects of ARE supplementation on the gut microbiota of individuals diagnosed with several types of cognitive impairment. Although the findings did not reveal statistically significant differences in overall alpha (Chao1, Shannon, and Simpson indices) and beta (Bray-Curtis and Jaccard indices) diversity measures, increases, and decreases in certain microbial groups with potential biological significance were identified. The genus-level changes observed in microbiota composition were not statistically significant and should therefore be interpreted not as definitive findings but as exploratory, hypothesis-generating observations.

In our study, the decrease in *Coriobacteriia* following ARE supplementation was consistent with findings from previous studies^76^.

In the literature, *Alistipes* has been reported to exhibit anti-inflammatory effects and support gut barrier functions^77^. Additionally, the increase in *Alistipes* has been associated with impacts on amino acid metabolism, particularly in modulating tryptophan derivatives, which play a role in regulating the gut-brain axis. This process can have significant effects on neurotransmitter production and neuronal activity^78^. The observed increase in *Alistipes* may indicate the potential neuroinflammation-reducing effects of ARE supplementation. Our findings suggest that the rise in *Alistipes* following ARE use may have positive implications for immune modulation and neurological health.

Polyphenol-based supplements have been reported in the literature to support the growth of gram-negative bacteria, with certain species within the *Enterobacteriaceae* family playing a role in flavonoid metabolism^79^. Specifically, some bacteria in the *Enterobacteriaceae* family can metabolize flavonoids to produce beneficial metabolites such as SCFAs, a process that enhances the gut microecosystem’s overall capacity to utilize bioactive compounds^80^.

However, the increase in *Enterobacteriaceae* has also been highlighted for its potential negative impact on gut health due to the presence of pathogenic species. For instance, species like *Escherichia coli* are known to be associated with inflammatory bowel diseases (IBD) and metabolic syndrome^79^. Moreover, an increase in *Enterobacteriaceae* can disrupt the balance of the microbial ecosystem, leading to dysbiosis, a condition linked to increased gut permeability and systemic inflammation.

Metabolites produced during flavonoid metabolism have been suggested in the literature to regulate gut-fiber metabolism and support energy homeostasis^81^. Furthermore, the increase in *Enterobacteriaceae* has been noted as a potential mechanism through which anthocyanins reshape the gut microbiota^77^. Positive effects of this increase include the enhancement of flavonoid metabolism and the optimization of immune responses. In our findings, the rise in *Enterobacteriaceae* following ARE supplementation aligns with and supports the objectives of our study.

*Streptococcus thermophilus* is known for its probiotic properties and its role in supporting gut health through lactic acid production^82^. Although alpha and beta diversity metrics did not reveal statistically significant changes at the community level, a 248% increase in *Streptococcus thermophilus* may still indicate biologically relevant shifts with potential functional implications. According to the literature, this species is recognized for its ability to maintain pH balance through lactic acid production, contributing to gut health^83,84^. The increase in *Streptococcus thermophilus* may align with the prebiotic effects of anthocyanins, which support specific microorganisms. This increase could enhance gut barrier function, reduce inflammation, and contribute to overall digestive health. Furthermore, it has been suggested that increased lactic acid production by this species may support the production of short-chain fatty acids (SCFAs), thereby improving energy metabolism. The rise in SCFAs may positively affect neuronal energy metabolism, ultimately improving cognitive functions^85^.

According to our findings, although the genus *Roseburia* showed a reduction following ARE supplementation, the change was not statistically significant. *Roseburia* is a butyrate-producing bacterium known for its anti-inflammatory properties^81^, suggesting that its response to ARE supplementation may be influenced by specific physiological factors. This potential sensitivity warrants further investigation through larger studies or experiments conducted under different conditions.

Additionally, a 49% reduction was observed in the genus *Hungatella*, which has recently gained attention due to its association with severe diseases. This bacterium has been linked to sporadic cases of bacteremia, fatal septicemia in adult patients, and severe COVID-19 cases^86,87^. The observed reduction in *Hungatella* may indicate a beneficial anti-inflammatory effect, potentially contributing to the overall impact of ARE supplementation on gut microbiota composition.

The 131% increase in the *Erysipelotrichaceae* family observed in our study may contribute to supporting gut health. The literature indicates that this family may reduce gut inflammation through the production of SCFAs^84^. An increase in SCFAs can improve energy metabolism, potentially exerting positive effects on both metabolic and neurological health^88^. However, the increase in *Erysipelotrichaceae* has also been associated with dysbiosis in some individuals, suggesting that its effects may vary depending on individual differences^84,89^. Furthermore, this increase may play a role in regulating lipid metabolism. Studies have reported that *Erysipelotrichaceae* can influence cholesterol metabolism and improve lipid profiles^83,90^.

The observed increase (133%) in *Flavonifractor plautii* is consistent with findings reported in the literature, even though it did not surpass the threshold for statistical significance. This species has been documented to support antioxidant activity and enhance gut barrier functions through polyphenol metabolism^91^. The increase in *Flavonifractor* may contribute to the reduction of oxidative stress and the optimization of immune responses via antioxidant mechanisms.

However, some studies have suggested that an increase in *Flavonifractor* could also be associated with inflammatory effects^83^. The rise in the *Flavonifractor* genus following ARE supplementation indicates that polyphenols may act as a substrate promoting the growth of this species. The impact of *Flavonifractor* on metabolic pathways has been linked to the conversion of flavonoid derivatives into smaller phenolic compounds, a process associated with reduced gut inflammation. Moreover, this metabolic transformation has been suggested to enhance mitochondrial energy production and play a critical role in reducing oxidative stress^92^.

Our study found that while ARE supplementation did not significantly alter overall gut microbiota diversity in individuals with cognitive impairment, it influenced specific microbial groups with potential neurological implications. Notable changes included a decrease in *Coriobacteriia*, which supports gut integrity, and an increase in *Alistipes*, associated with reduced neuroinflammation and gut-brain axis modulation. The rise in *Enterobacteriaceae* suggests enhanced flavonoid metabolism but also raises concerns about potential dysbiosis. Additionally, increases in *Streptococcus thermophilus*, *Erysipelotrichaceae*, and *Flavonifractor plautii* may contribute to gut health and metabolic support, while the reduction in *Hungatella* suggests a possible anti-inflammatory effect. However, the fact that all of our participants were elderly individuals residing in a long-term care institution represents another important factor to consider. As demonstrated in previous studies, such as the ElderMet cohort, aging is typically associated with reduced gut microbiota diversity and alterations in metabolic capacity^93–95^. The longer individuals remain in institutional care, the more pronounced these changes tend to become, potentially limiting their ability to metabolize dietary components such as anthocyanins. This age-related decline in microbiota diversity and functionality may partly explain the lack of statistically significant findings in our study. These findings highlight a potential association between anthocyanin intake and limited genus-level shifts in gut microbiota, which may be linked to cognitive health; however, further research is necessary to clarify these relationships.

Beyond compositional shifts, some microbial taxa exhibited trends toward associations with EEG metrics, suggesting possible links between gut microbiota and neural complexity. Among these, *Coriobacteriia* displayed a notable correlation with FD, as highlighted in Table S1. Members of this class are known to influence neuroinflammation and synaptic activity through polyphenol metabolism and bile acid transformation^96,97^. Given that FD typically decreases in cognitive impairment^65,69^, this positive association suggests that *Coriobacteriia* may contribute to preserving signal complexity. Importantly, despite its reduced overall abundance, *Coriobacteriia* retained functional correlations with EEG metrics. Together with *Turicibacteraceae* and *Dysosmobacter welbionis*, which are implicated in SCFA production and inflammatory modulation^98,99^, these taxa may help explain the observed improvements in EEG connectivity and complexity in the beta–gamma frequency bands. These findings underscore the need to consider not only compositional shifts but also functional correlations when evaluating microbiota–brain interactions.

This study indicates that ARE supplementation may be associated with alterations in both functional EEG connectivity and gut microbiota composition, suggesting a possible neuroprotective interaction via the gut-brain axis, though causality remains to be established. The gut-brain axis links gut microbiota, neurotransmitter pathways, and immune regulation to brain function, influencing cognition and neurodegeneration^100–102^. Given the role of gut dysbiosis in cognitive decline, our findings suggest that anthocyanins may enhance brain function through gut microbial shifts.

Although SCFAs such as butyrate, acetate, and propionate are known to cross the blood–brain barrier, modulate neuroinflammation, and regulate synaptic activity^103–105^, it is important to emphasize that SCFA levels were not directly measured in this study. Moreover, 16S rRNA sequencing provides limited taxonomic resolution and does not allow robust functional or metabolic inference. Therefore, any potential role of SCFAs in mediating the observed effects should be regarded as speculative. The observed changes in *Streptococcus thermophilus* and *Erysipelotrichaceae* may be biologically interesting trends, but given the lack of statistical significance and absence of direct metabolite data, they should be interpreted cautiously and considered as hypothesis-generating observations for future studies. While speculative, these microbial shifts could indicate an environment conducive to SCFA-related activity, which may in turn support neural function. This interpretation aligns with the observed increase in EEG connectivity measures (iCOH, GE, LE) across alpha, beta, and gamma bands, although direct evidence of SCFA modulation remains to be established.

Anthocyanins are not directly fermented into SCFAs; their potential prebiotic effects occur indirectly by modulating the composition of the gut microbiota and thereby influencing the fermentation of dietary fibers^106,107^. In addition, anthocyanins are metabolized by gut microbes into simpler phenolic acids, which enter the circulation via the hepatic portal vein, undergo conjugation and other processing in the liver, and some of which have the potential to cross the blood–brain barrier, thereby directly influencing brain activity or acting through peripheral immune signaling pathways^106,108,109^. Although SCFA levels were not directly measured in this study, we observed changes in bacterial taxa known to be involved in SCFA metabolism. Specifically, an increase in *Streptococcus thermophilus*—a species associated with SCFA production—may imply a shift toward greater SCFA biosynthetic potential^103,105^. Similarly, the increased relative abundance of *Erysipelotrichaceae*, a family implicated in SCFA production and lipid metabolism, supports this inference^110,111^. While these microbiota shifts do not confirm elevated SCFA concentrations, they suggest a microbial environment more conducive to SCFA-related activity. Notably, the observed increase in EEG-based connectivity metrics (iCOH, GE, LE) in the alpha, beta, and gamma bands may reflect improved neural efficiency potentially associated with such microbial changes, although this interpretation remains speculative without direct metabolomic validation^103,105,112^. However, shotgun metagenomic sequencing was not applied in this study; this method could provide critical contributions in the future by enabling the detection of SCFA-related genes and offering higher taxonomic and functional resolution^113^. Due to financial constraints, this approach could not be included in our study and should be noted as a limitation.

The gut microbiota regulates tryptophan metabolism, influencing serotonin (5-HT) and kynurenine pathways^114,115^. The increase in *Alistipes*, a genus involved in tryptophan metabolism and immune regulation, suggests that anthocyanins may optimize neurotransmitter synthesis, potentially explaining the rise in EEG complexity measures (QSE, QG) in the gamma frequency band, linked to cognitive processing^116,117^.

Anthocyanins can alter gut microbiota composition by increasing species involved in polyphenol metabolism^110,111^. The observed increase in *Enterobacteriaceae* suggests microbial shifts that may enhance anthocyanin metabolism, but some species within this family are also linked to gut dysbiosis and neuroinflammation^102,118,119^. This dual effect underscores the complexity of microbiota-driven cognitive modulation.

*Roseburia*, a key butyrate-producing bacterium, showed a slight decrease. Since butyrate is essential for neuronal metabolism and synaptic plasticity, this reduction suggests an individualized microbial response to anthocyanins^112^.

A reduction in *Hungatella*, a bacterial genus linked to systemic inflammation, suggests that anthocyanins may mitigate gut-derived neuroinflammatory responses^102,118^. Since neuroinflammation is associated with disrupted EEG connectivity, this shift may contribute to the observed EEG improvements. It should also be acknowledged that microbiome-related interventions, including those with polyphenols, are increasingly subject to the responder–non-responder phenomenon^120,121^. Individual differences in baseline microbiome composition and metabolic potential may explain variability in responses to ARE supplementation observed in our study. Future studies should address this heterogeneity through approaches such as pre-screening of microbial composition or combining ARE with selected probiotics to enhance the consistency of microbiota-mediated effects.

These findings suggest that anthocyanins enhance cognition via microbiota-driven pathways by increasing SCFA production, optimizing neurotransmitter metabolism, and reducing neuroinflammation. The observed increases in EEG connectivity and complexity across alpha, beta, and gamma bands indicate a potential restoration of disrupted neural networks associated with cognitive impairment. Future research should further explore the causal relationships between gut microbial shifts and EEG changes, particularly in the context of long-term anthocyanin supplementation.

## Methods

### Patient selection

This study examines the long-term effects of ARE consumption on elderly individuals diagnosed with subtypes of cognitive impairments. To ensure consistency in living conditions, participants were selected from residents of the Darülaceze Senior Center, operated by the Istanbul Metropolitan Municipality (Istanbul, Turkiye). All participants included in this study were of Turkish ethnicity. While 86 elderly individuals were initially enrolled, 36 were excluded from the final analysis for various reasons. These included the emergence of new health problems that rendered participation unsafe, hospitalization or acute medical complications, non-adherence to the intervention protocol, and discontinuation of the ARE supplement. Reasons for non-compliance included difficulty swallowing or refusal to continue supplementation. Additionally, some participants withdrew their consent or were unable to complete the study assessments. As a result, data from 50 participants were included in the final analysis. Their detailed demographic and clinical data are presented in Table 3.

**Table 3:** Demographic and Clinical Characteristics of Study Participants.

Subject number	50
Laterality	90% Right-handed & 10% Left-handed
Age (Mean ± SD)	$83.14 \, (\pm 9.16)$ years
Gender	62% men & 38% women
Literacy rate	40%
BMI (Mean ± SD)	$25.95 \, (\pm 5.37) \, \mathrm {kg/m^2}$
Diagnosis	90% Dementia & 10% AD
TDA (Mean ± SD)	$5.14 \, (\pm 4.19)$ years

(SD: Standard Deviation, BMI: Body Mass Index, AD: Alzheimer’s disease, TDA: Time from Diagnosis to Assessment).

Notably, studies investigating the effects of nutrition-based interventions on cognitive function using EEG typically employ sample sizes ranging from 25 to 60 participants. According to a power analysis conducted using G*Power 3.1 software, a sample size of 50 provides over 80% statistical power to detect a medium effect size (Cohen’s $d \approx 0.5$) at a significance level of $\alpha = 0.05$. Similar sample sizes have been reported in the literature for studies examining the impact of nutraceutical interventions on EEG signals^122,123^.

EEG recordings were analyzed for all 50 participants, while gut microbiota analysis was conducted on a subsample of 13 individuals, as only these stool samples yielded DNA of sufficient quality and quantity for downstream analysis. Although all samples were collected and processed under identical conditions, unsuccessful extractions could not be repeated due to the technical demands and high cost associated with 16S rRNA sequencing and downstream bioinformatics. Thus, the final subsample was primarily determined by DNA quality limitations, with financial and technical constraints acting as secondary factors that prevented further expansion. Importantly, these 13 individuals were not selected based on clinical or demographic characteristics but were simply those meeting the DNA quality threshold, thereby minimizing the risk of selection bias. Nevertheless, this sample size falls within the typical range for pilot or exploratory human studies investigating diet–microbiota interactions, where group sizes often do not exceed 15 participants. Furthermore, because the study design focused on within-subject (pre–post) comparisons, such sample sizes can still yield meaningful insights^124^. Further details on this subgroup are provided in Table 4.

**Table 4:** Demographic and Clinical Characteristics of the Gut Microbiota Subgroup.

Subject number	13
Laterality	85% Right-handed & 15% Left-handed
Age (Mean ± SD)	$84.99 \, (\pm 5.92)$ years
Gender	54% men & 46% women
Literacy rate	46%
BMI (Mean ± SD)	$25.81 \, (\pm 5.31) \, \mathrm {kg/m^2}$
Diagnosis	85% Dementia & 15% AD
TDA (Mean ± SD)	$7.92 \, (\pm 6.41)$ years

(SD: Standard Deviation, BMI: Body Mass Index, AD: Alzheimer’s diseases, TDA: Time from Diagnosis to Assessment).

Diagnosis of cognitive impairment subtypes was made by neurologists and psychiatrists at Darülaceze, based on the criteria outlined in the International Classification of Diseases, 10th Revision (ICD-10)^125^. As part of routine clinical evaluations conducted before the study, the attending psychiatrists administered the Mini-Mental State Examination (MMSE), and indications of cognitive impairment were recorded in participants’ medical files. However, the research team did not have access to individual MMSE scores. All individuals included in the study were over 65 years of age. Patients with epilepsy, cardiovascular disease, cancer, significant psychiatric diagnoses affecting cognitive functions, neurodegenerative diseases, a history of head trauma or stroke, hydrocephalus, or tumors were excluded.

Informed consent was obtained from all participants and/or their legal guardians, who signed consent forms acknowledging their understanding and approval of the study objectives. Furthermore, the study protocol adheres strictly to the principles outlined in the Declaration of Helsinki and was approved by the Ethical Committee of Istanbul Nişantaşı University. The study was also approved by the Istanbul Darülaceze Branch Directorate, Health Department of the Istanbul Metropolitan Municipality, with decision number 1534, dated 13.12.2022.

### Study design

Individuals consumed 125 ml of drinking water containing 300 mg of anthocyanin extract daily for 12 weeks while maintaining their usual diet. No specific dietary intervention was introduced; however, all participants received standardized meals prepared by the institutional kitchen of the Istanbul Darülaceze Directorate. As such, dietary intake was relatively uniform across individuals, minimizing nutritional variability.

The 300 mg/day dosage was selected based on previous clinical studies and safety evaluations. For instance, Do et al. (2021) reported that an 8-week supplementation of 201 mg/day anthocyanins significantly reduced TNF-$\alpha$ levels in individuals with mild cognitive impairment, supporting their role in modulating neuroinflammation^126^. Similarly, Aarsland et al. (2023) demonstrated that a 24-week supplementation of 320 mg/day anthocyanins led to significant improvements in episodic memory among elderly individuals^127^. Wood et al. (2023) also found that a daily dose of 302 mg anthocyanins improved episodic memory and executive function in individuals at risk for cognitive decline^128^. Reported effective doses in the literature range from 10 to 425 mg/day^9^, and the acceptable daily intake (ADI) based on animal studies is approximately 2.5 mg/kg/day^129,130^. While this ADI applies to healthy individuals, higher doses are often used in clinical contexts due to increased physiological demands^131,132^. Considering the enhanced bioavailability and stability of our formulation, the 300 mg/day dose was deemed both safe and potentially effective.

Use of cognitive enhancers was permitted during the study. The majority of participants (n=41) were on stable regimens of commonly prescribed agents, including Donepezil, Rivastigmine, Memantine, Ginkgo biloba extract, or Piracetam. These medications had been initiated at least eight months before the start of the study (mean duration: $3.6 \pm 2.9$ years), and no changes in type or dosage were allowed during the intervention period. Similarly, the use of SSRIs (e.g., escitalopram, sertraline), statins (e.g., atorvastatin, rosuvastatin), and antihypertensives (e.g., amlodipine, ramipril, metoprolol) was common among participants (n=35). These treatments had been initiated between 9 months and 12 years before the first EEG recording (mean duration: $3.06 \pm 2.34$ years) and remained unchanged during the intervention, thereby minimizing potential confounding effects. Their influence was therefore considered stable and unlikely to introduce temporal bias within the study window. AREs were administered by nursing staff each morning after breakfast, and participants were monitored throughout the intervention. Individuals who discontinued supplementation for more than one week were excluded from the final analysis.

EEG recordings were conducted before and after the 12-week term, during resting-state conditions with participants seated in a quiet, dimly lit room. Recordings were performed with eyes closed, and each session lasted a minimum of 30 minutes to ensure robust analysis of resting-state brain activity.

Stool samples were collected before and after the 12-week period for microbiota analysis, with assistance from caregivers. At baseline, stool samples were collected within 2 days prior to the start of the intervention, and post-intervention samples were obtained within 2 days after completion of the 12-week period. This timeframe was applied to minimize variability in microbiota composition due to temporal differences in sampling. For bedridden participants, samples were obtained from diapers; for those who were independently mobile, samples were collected at the toilet. To ensure sample integrity, the time between defecation and collection was limited to a maximum of 15 minutes. Caregivers and participants were instructed accordingly to avoid exceeding this duration. Samples were immediately transferred into appropriate containers, stored at $-80^{\circ }\text {C}$, and transported under cold-chain conditions to the laboratory for microbiota analysis.

### Extraction and analysis of anthocyanins

#### Extraction and purification of anthocyanins

The extraction of anthocyanins was conducted with modifications to the method previously described by Eichhorn et al.^133^ and Hillebrand et al.^134^. Initially, black carrot (Daucus carota L. spp. sativus var. Atrorubens Alef.) was finely chopped, and approximately 100 g of the material was immersed in 1 L of boiling water ($100^{\circ }\text {C}$) for 3 minutes. Subsequently, an equal volume of a solution containing 1% HCl was added. The mixture was then cooled to $0^{\circ }\text {C}$ and extracted using ultrasonication for 30 minutes. The extraction solution was centrifuged to collect the supernatant.

A D-101 resin system was first loaded into the column for conditioning in the purification step. During the adsorption phase, the supernatant from centrifugation was passed through the column 4−5 times. The column was then washed with distilled water to remove sugars, proteins, organic acids, and other components. In the desorption phase, an ethanol-water mixture was applied to the column to elute the anthocyanins. Finally, ethanol was removed from the collected mixture using a rotary evaporator, and the remaining aqueous phase was lyophilized to obtain a powdered form.

#### Analysis of anthocyanins by HPLC-DAD

Anthocyanins in the lyophilized black carrot extract were analyzed using a modified HPLC method based on Vagiri et al.^135^. A Shimadzu LC-20A HPLC system (Kyoto, Japan) was employed, UV-Vis photodiode array detector (SPD-M20A), degasser (DGU-20A), comprising a pump (LC-20AT), and column oven (CTO-10AS). The extract was prepared at various concentrations, and filtered through a $0.45\,\upmu \text {m}$ membrane, and the mobile phases were similarly filtered and degassed.

HPLC analyses were performed with a $5\,\upmu \text {m}$ Inertsil ODS-3 reverse-phase column (250 x 4.6 mm; GL Sciences). The sample injection volume was set to $20\,\upmu \text {L}$, and the column oven temperature was maintained at $25^{\circ }\text {C}$. The mobile phases employed were A (water/formic acid, 93/7) and B (acetonitrile/methanol/water, 90:5:5), with a 1.2 mL/min gradient flow rate. The gradient program was as follows: 0 to 2 minutes, 8% B; 2 to 21.5 minutes, 16% B; 21.5 to 30 minutes, 18% B; and re-equilibration with 8% B for 5 minutes. Chromatograms for anthocyanins were recorded at 520 nm using the UV-Vis detector. The total anthocyanin content was expressed as mg cyanidin-3-O-glucoside equivalents per gram of sample (mg C3GE/g sample), and all data were analyzed using LCsolution software. The results of this analysis are presented in Table 5.

**Table 5:** Quantitative Evaluation of Bioactive Compounds and Antioxidant Activity in Purified Black Carrot Extract.

**Sample**	**TAC**	**TPC**	**TEAC**
	(mg C3GE*/125 mL)	(mg GAE**/125 mL)	($\mu$mol TRE***/125 mL)
Purified Black Carrot Extract	$300 \pm 2$	$915 \pm 10$	$7800 \pm 70$

*TAC: Total Anthocyanin Content (expressed as mg cyanidin-3-glucoside equivalents per 125 mL), **TPC: Total Phenolic Content (expressed as mg gallic acid equivalents per 125 mL), ***TEAC: Trolox Equivalent Antioxidant Capacity (expressed as $\upmu$mol Trolox equivalents per 125 mL).

#### Total phenolic content analysis

The total phenolic content of the black carrot extract was determined using the Folin-Ciocalteu method as described by Singleton et al.^136^. In brief, 0.2 mL of the sample at various concentrations was mixed with 1.5 mL of 10% Folin-Ciocalteu reagent and allowed to stand for 5 minutes. Subsequently, 1.2 mL of 7.5% (w/v) sodium carbonate (Na2CO3) was added to the mixture, and the mixture was thoroughly stirred to ensure complete homogenization. After incubating at room temperature for 60 minutes, the absorbance was measured at 765 nm using a Shimadzu UV-1800 spectrophotometer. Analyses were performed in triplicate, and the calibration curve was generated using gallic acid in the range of 0–250 ppm. Results were expressed as milligrams of gallic acid equivalents per gram of extract (mg GAE/g extract). The results of the analysis are shown in Table 5.

#### Total antioxidant capacity analysis (CUPRAC)

The antioxidant capacity of the black carrot extract was assessed using the CUPRAC (Cupric Ion Reducing Antioxidant Capacity) method as described by Apak et al.^137^. In summary, 1 mL of 10-2 M CuCl2, 1 mL of 7.5x10-3 M neocuproine, and 1 mL of 1 M NH4Ac were mixed in a test tube. Subsequently, the sample (or standard) solution was prepared at various concentrations (x mL) and H2O (1.1-x mL) was added to the initial mixture to reach a final volume of 4.1 mL. This mixture was incubated at room temperature for 30 minutes. Finally, the absorbance was measured at 450 nm using a Shimadzu UV-1800 spectrophotometer (Kyoto, Japan). The calibration curve was generated using Trolox in the 10-$100\,\upmu$mol/L range. The results of this analysis are presented in Table 5.

### EEG recording and processing

#### EEG recording and preprocessing

EEG recordings in this study were conducted with a 10–20 system gel cap. Each recording session maintained a consistent sampling rate of 500 Hz and employed a finite impulse response (FIR) band-pass filter ranging from 1 to 45 Hz to capture relevant neural activity while minimizing noise interference. To ensure data integrity and quality, mastoid referencing was selected as the preferred referencing method for all recordings.

In the initial preprocessing stage, the raw EEG dataset underwent several key steps to enhance interpretability and facilitate subsequent analysis. Firstly, the dataset was re-referenced using the surface Laplacian method with the New Orleans configuration, a technique known for its effectiveness in decreasing volume conduction effects on coherence analysis^138^. This re-referencing procedure was implemented using a MATLAB function developed by Cohen^139^, specifically tailored for functional connectivity calculations. Additionally, to optimize the computation of various metrics such as FD, QSE, QG, and VG, another re-referencing step was performed using the Common Average Referencing (CAR) method^140,141^. This method, implemented via a predefined MATLAB function^142^, serves to mitigate spatial biases and enhance the comparability of EEG signals across channels.

After re-referencing stages, we integrated a predefined function incorporating the Artifact Subspace Reconstruction (ASR) method to further refine the data by eliminating noisy segments from the EEG traces^143^. The ASR function utilized in this study is integrated into the EEGLAB toolbox^144^, a widely adopted toolbox for EEG signal processing and analysis. Technical details regarding the ASR method are elaborated in^145^, providing insights into its underlying principles and empirical validation.

Following the application of noise removal, we employed the Wavelet-enhanced Independent Component Analysis (wICA) method to automatically identify and suppress components likely to represent noise sources within the EEG data. This method, renowned for its effectiveness in separating neural signals from various forms of noise, was implemented using a dedicated MATLAB toolbox^146^. As a result of the combined wICA and ASR preprocessing steps, EEG signal traces of high quality, each with a minimum duration of 20 minutes, were obtained, ensuring robustness in subsequent analysis.

#### EEG quantitative analysis

We analyzed EEG signals using a diverse array of quantitative metrics to assess changes in brain activity and connectivity over three months of ARE consumption. We segmented signals into seven frequency bands: Delta (1–3 Hz), Theta (3–7 Hz), Alpha (7–12 Hz), Beta I (12–18 Hz), Beta II (18–24 Hz), Beta III (24–30 Hz), and Gamma (30–45 Hz). We calculated each metric for every frequency band and the entire spectrum (1–45 Hz).

In this study, functional connectivity across different frequency bands was computed using the iCOH due to its efficacy in mitigating the confounding effects of volume conduction compared to standard coherency^147^. Coherency ($C_{ij}(f)$) and its associated components were calculated as detailed in the Supplementary 1.1. These calculations utilized a Hanning window of 1-second length and a 50% segment shift. Imaginary coherence, the imaginary part of the absolute value of coherency, was then computed across the mentioned frequency bands. iCOH values were computed for 21 channels, resulting in the analysis of each of the 210 electrode pairs.

GE serves as a fundamental metric in graph theory, quantifying functional integration. This metric offers valuable insights into the overall efficiency of information transfer and communication within the brain network^148,149^. The formula for GE is provided in see Supplementary 1.2.

LE, is a key network measure that reflects the average efficiency of information transfer among the neighbor nodes of a given node, providing insights into the local processing capabilities of the network^54^. The formula for LE is detailed in Supplementary 1.3.

The last coherence metric, T is a commonly employed metric to assess the likelihood of interconnectedness between indirectly linked nodes within a network^150^. The formula for T is provided in Supplementary 1.4.

For the calculation of the FD, QSE, QG, and VG measures, all recordings were downsampled from 500 to 200 Hz and divided into 8-second epochs, each containing 1,600 samples.

FD is a derived measure that represents the similarity and complexity of a signal, providing insights into its structural characteristics. In this study, the Katz FD algorithm was preferred due to its lower computational cost compared to the Higuchi algorithm^151^. As a result of this algorithm, FD takes values between 1 and 1.5. While 1 represents a straight line, the similarity and complexity of the signal increase towards 1.5, reaching the maximum point. The formula for Katz FD is provided in Supplementary 1.5.

QSE is one of the entropy calculation methods that quantifies the randomness and unpredictability in a time series. Higher entropy indicates more irregularity and uncertainty. Richman and Moorman^152^ proposed QSE to overcome the limitations of parameter dependence inherent in entropy calculation methods like Approximate Entropy (ApEn)^153^ and Sample Entropy (SampEn), which have been widely used for analyzing physiological signals^154^. The calculation of QSE is given in Supplementary 1.6.

QG and VG analyses transfer time series into complex graph domains. In this way, the properties of complex graph theory are utilized to extract information about the signal’s complex and dynamic structure. The time series is divided into quantiles in QG analysis based on its amplitude. Then, a network model is created using the neighborhood relationships of these quantiles. The topology and certain properties of the created network model are informative about the time series^155^. In this study, one of these topological properties, the average hop length ($\Delta (k)$), is considered. Calculations are given in the Appendix Supplementary 1.7. In the VG method, a network model is constructed by determining whether points in a given time series satisfy the visibility criteria, meaning they can see each other without obstructions. In this way, fractal structures can mapped within the time series into scale-free graphs. Calculations of the algorithm^156^ used are included in Appendix Supplementary 1.8. For the comparison of all these metrics before and after ARE consumption, the Wilcoxon signed-rank test was applied using Python while p-value $<0.05$ considered statistically significant. To control the FDR, *p*-values were further adjusted using the Benjamini–Hochberg procedure. FDR correction was performed individually for each nonlinear EEG metric, and in line with the exploratory nature of the study, significance was considered at $q < 0.10$.

### DNA isolation and optimization

DNA isolation was performed using the Quick-DNA^TM^ Fecal/Soil Microbe Miniprep Kit (Zymo Research, Irvine, CA, USA)^157^, based on the manufacturer’s protocol. However, due to the complex nature of stool samples, several optimization steps were experimentally tested and applied. Through these trials, the conditions that yielded the highest DNA yield and purity were optimized. To enhance cell lysis efficiency, the vortexing duration was extended to 1 hour, and zirconium beads were used in addition to ceramic beads. The centrifugation time was adjusted to 2 minutes, the supernatant volume was set to 600 $\mu$L, and the Genomic Lysis Buffer volume was set to 1,800 $\mu$L. The column loading step was repeated twice, and the DNA washing duration was extended to 2 minutes. In the elution step, the Elution Buffer was pre-heated to $60^{\circ }\text {C}$, and a final volume of 30 $\mu$L was used.

The subsequent steps of DNA isolation were performed without any modifications. The extracted DNA was stored at $-20^{\circ }\text {C}$ until further downstream applications such as PCR and sequencing. DNA degradation and contamination were assessed using a 1% agarose gel, while purity ratios (OD260/OD280, OD260/OD230) were evaluated with a Qubit fluorometer (Invitrogen). DNA concentration was measured with the Qubit^®^ dsDNA Assay Kit on the Qubit^®^ 2.0 fluorometer, using 1 $\mu$L of sample in the High Sensitivity dsDNA program (Life Technologies, CA, USA). DNA samples with an OD value of approximately 5.0 and a yield of more than 1 $\mu$g were used for amplicon generation prior to library preparation. As a result of these systematic optimizations, the extracted DNA achieved optimal values in both concentration and purity, providing a high-quality starting material that enhanced the reliability of subsequent molecular analyses.

### Analysis of the gut microbiome and its changes

This study utilized a robust sequencing and bioinformatics pipeline to characterize the richness and evenness of bacterial taxa, their phylogenetic relationships, and the dissimilarities between pre- and post-intervention states. Amplicon libraries were prepared using the 16S Barcoding Kit 1–24 (SQK-16S024, Oxford Nanopore Technologies, UK)^158^, which contains primer sets targeting near full-length 16S rRNA gene regions^159^. Barcode sequences from Oxford Nanopore Technologies were incorporated at the $5^{\prime }$ ends of the primers. Primer pairs targeting the V1–V9 regions of the 16S rRNA gene were used, with the following sequences: forward primer $5^{\prime }$-TTTCTGTTGGTGCTGATATTGC-AGRGTTTGATYHTGGCTCAG-$3^{\prime }$ and reverse primer $5^{\prime }$-ACTTGCCTGTCGCTCTATCTTC-TACCTTGTTAYGACTT-$3^{\prime }$. PCR amplification was then performed by adding 10 $\mu$l of each 16S Barcode to the respective sample-containing tubes, with an initial denaturation at $95^{\circ }\text {C}$ for 1 min, followed by 25 cycles of denaturation at $95^{\circ }\text {C}$ for 20 s, annealing at $55^{\circ }\text {C}$ for 30 s, and extension at $65^{\circ }\text {C}$ for 2 min, with a final extension at $65^{\circ }\text {C}$ for 5 min and a hold at $4^{\circ }\text {C}$. Amplification was carried out using the LongAmp^®^ Hot Start Taq 2X Master Mix (New England Biolabs, M0533S), supplied within the 16S Barcoding Kit, eliminating the need for any additional external proofreading polymerase. The resulting amplicons were verified by agarose gel electrophoresis, purified, and quantified spectrofluorometrically. Barcoded DNA samples were pooled and loaded onto an R9.4.1 (FLO-MIN106) flow cell, and sequencing was carried out on the Mk1C™ device using MinKNOW™ software (Oxford Nanopore Technologies, UK)^160^.

Raw sequencing data were converted to FASTQ format using Guppy^161^, and operational taxonomic units (OTUs) were identified using BLAST^162^. Phylogenetic and taxonomic analyses were conducted with bioinformatics tools such as Qiime2^36^ and Mothur^163^, while visualizations were generated using Python.

The gut microbiota within the bacterial domain was analyzed using comprehensive metrics. Alpha ($\alpha$) diversity, which measures community richness, was quantified with the Chao1 index^164^, while community evenness was evaluated using the Shannon index^165^ and the Simpson index^166^. Beta ($\beta$) diversity, which assesses community dissimilarity, was calculated using the Jaccard^167^ and Bray-Curtis^168^ indices. Principal Coordinate Analysis (PCoA)^169^ was used to visualize differences in community structure.

To assess statistical changes in alpha diversity between pre- and post-intervention conditions, the Kruskal-Wallis test was applied. Pairwise PERMANOVA was used to analyze differences in beta diversity metrics. Species-level comparisons between pre- and post-intervention conditions were conducted using the Wilcoxon signed-rank test. A p-value of $<0.05$ was considered statistically significant for all tests.

### Correlation analysis between EEG and gut microbiota

To investigate potential gut–brain axis interactions, correlation analyses were performed specifically between changes in EEG metrics and relative abundances of gut microbial taxa. For each participant with available microbiome data, pre–post differences were calculated separately for each nonlinear EEG feature (FD, QSE, QG, VG) across frequency bands and for microbial taxa that showed relative abundance shifts following ARE intake. Kendall’s tau correlation was employed to assess associations, given its suitability for small sample sizes and non-parametric distributions. FDR controlled using the Benjamini–Hochberg procedure. As an exploratory step, microbial–EEG feature pairs with uncorrected $p < 0.001$ were additionally evaluated with Spearman’s rank correlation, and these results are reported in the Supplementary Materials (Table S1).

## Conclusion

This study suggests that daily ARE supplementation for 12 weeks may be associated with changes in neural activity and gut microbiota composition in individuals with cognitive impairment. Our findings indicate increased EEG connectivity, particularly in alpha, beta, and gamma frequency bands, alongside notable shifts in microbial populations involved in SCFA production, tryptophan metabolism, and neuroinflammation regulation. While the gut microbiota analysis did not reveal statistically significant changes in overall diversity, specific microbial groups exhibited alterations that align with potential neuroprotective mechanisms.

The observed increases in functional connectivity metrics, such as iCOH, GE, LE, and T, suggest enhanced neural communication following ARE consumption. Increases in QSE and QG in higher frequency bands further support the hypothesis that anthocyanins may improve cognitive resilience by enhancing neural complexity. Conversely, decreases in FD and VG, particularly in delta and theta bands, point to potential alterations in low-frequency oscillatory dynamics.

Microbiota findings highlighted an increase in *Alistipes* and *Streptococcus thermophilus*, suggesting possible improvements in neurotransmitter synthesis and gut-brain axis interactions. The rise in *Erysipelotrichaceae* and *Flavonifractor* supports the notion that anthocyanins may influence SCFA metabolism and gut barrier function. Additionally, the decrease in *Hungatella*, a genus linked to inflammation, aligns with the observed EEG patterns associated with reduced neuroinflammatory activity.

These results underscore the potential of ARE supplementation as a dietary factor that may influence cognitive health through gut-brain axis interactions; however, further research is needed to establish causality. However, the non-significant changes in overall microbiota diversity and the contrasting trends observed in nonlinear EEG metrics highlight the need for further research with larger cohorts and extended supplementation periods. Future investigations should aim to elucidate the long-term effects of anthocyanins on cognitive function and their underlying mechanisms through microbiota-driven pathways. Ultimately, this study contributes to the growing body of evidence supporting the potential role of polyphenols in promoting brain health during aging and cognitive impairment.

## Limitations and future directions

This study has several limitations. The 12-week supplementation period provided insights into EEG and gut microbiota changes; however, the effects on specific neurocognitive disorders remain unclear due to the heterogeneous participant group, which included individuals diagnosed with dementia and Alzheimer’s disease. An additional limitation is the lack of systematically recorded MMSE scores. Although cognitive impairment was routinely assessed by psychiatrists and diagnoses were available, individual MMSE scores were not accessible to the research team. Moreover, clinical diagnosis was not included as a covariate due to the small and unbalanced diagnostic subgroups. Future studies should incorporate both MMSE scores and diagnostic information as covariates to improve analytic precision and better account for variability in cognitive functioning.

Stool samples were collected only once before and after the intervention due to financial and logistical constraints, limiting our ability to monitor temporal fluctuations and account for individual variability in microbial composition. In addition, the number of microbiota samples that yielded high-quality sequencing data was limited (n=13), which further constrains the statistical power and generalizability of these findings, and therefore the results should be interpreted as exploratory. Factors such as diet, age, and general health status—known to influence gut microbiota—could not be fully controlled under this restricted sampling design. Another limitation of this study is that the participants were elderly individuals residing in an institutional care setting. This condition may lead to reduced gut microbiota diversity and metabolic capacity, thereby limiting the potential for anthocyanin metabolism. Future studies should carefully consider the potential impact of age and institutional living conditions on gut microbiota composition and functionality. In addition, future research could explore a synbiotic approach by combining AREs with selected probiotic strains capable of metabolizing polyphenols. Such a strategy may enhance the bioavailability of anthocyanins, strengthen microbiota-mediated effects, and contribute to more effective modulation of the gut–brain axis. One limitation of this study is that habitual dietary intake was not systematically recorded. Although all meals provided during the intervention period were standardized, participants may still have varied in their total energy and nutrient intake. However, since all participants were institutionalized older adults residing in the Darülaceze Senior Center where meals are routinely standardized, the potential variability in dietary intake was likely minimized. Future studies should incorporate dietary records or food frequency questionnaires to better account for these potential confounders. Although environmental exposure was minimized by limiting the time stool samples remained in the diaper or toilet, short delays may still have introduced minor alterations in microbial profiles. Moreover, fecal samples, while practical and widely adopted, offer only an indirect representation of the gut microbiota and may not fully capture its compositional or functional complexity. The absence of direct measurements of microbial metabolites, such as SCFAs, precludes definitive mechanistic conclusions. It should also be noted that anthocyanins themselves are not directly fermented into SCFAs; their potential prebiotic effects are indirect, occurring through modulation of gut microbial composition which in turn can influence fiber fermentation. It is also possible that changes in metabolite profiles require longer follow-up periods to become detectable. In future studies, we aim to address these limitations by incorporating longitudinal sampling, dietary tracking, and refined collection protocols. We also intend to integrate 16S rRNA sequencing with targeted SCFA quantification, untargeted metabolomics, and plasma cytokine profiling to more comprehensively investigate the complex interplay between anthocyanin supplementation, gut microbiota, and brain function.

Although ARE supplementation was applied in this study, it was designed as a small-scale exploratory pilot project. Therefore, it was not registered in a clinical trial registry, as the primary objective was to assess feasibility and generate preliminary findings under institutional ethical approval. The absence of a control group also represents a key limitation, restricting the ability to draw definitive causal inferences. While our pre-post design enabled within-subject comparisons, baseline variability and external influences could not be fully controlled without a comparator arm. Future studies should include appropriate control conditions to enable more robust evaluation of intervention effects. Formal registration of future, larger-scale trials—ideally involving disorder-specific cohorts and randomized controlled designs, potentially integrating EEG with neuroimaging techniques—could provide clearer insights into gut-brain interactions in the context of cognitive impairment.

Finally, although the use of cognitive enhancers as well as the chronic use of other commonly prescribed medications in this cohort, including SSRIs, statins, and antihypertensives, remained stable throughout the intervention, their potential long-term effects on EEG and microbiota composition cannot be entirely excluded and should be taken into account in future controlled studies.

## Supplementary Information

Supplementary Information.

## Data Availability

The data that support the findings of this study are available from the corresponding author upon reasonable request.
