# Supplementary material for: Feeding the mind: preliminary insights into the effects of Anthocyanin-Rich Extract from black carrots on brain activity and gut microbiota in patients with cognitive impairments
Source: Sci Rep. 2025 Nov 27;15:45450. doi: 10.1038/s41598-025-29256-z (PMC12749660; doi:10.1038/s41598-025-29256-z)
Supplement: Supplementary file 1 — Supplementary Information. [file 41598_2025_29256_MOESM1_ESM.pdf]

Feeding the Mind: Preliminary Insights into the Effects of Anthocyanin-Rich Extract from Black Carrots on Brain Activity and Gut Microbiota in Patients with Cognitive Impairments

Aynur Muduroglu-Kirmizibekmez,[1,!], Alparslan Onder[2], Mustafa Yasir Ozdemir[2,3], Gamze Gurerk[4], Sevcan Aydin[4], Onder Yuksel Eryigit[5], and Ihsan Kara[6,*]

[1]Istanbul Nisantasi University, Department of Basic Science, Istanbul, 34398, Turkiye
[2]SANKARA Brain and Biotechnology Research Center, Istanbul, 34320, Turkiye
[3]Izmir Katip Celebi University, Department of Biomedical Engineering, Izmir, 35620, Turkiye
[4]Istanbul University, Division of Biotechnology, Biology Department, Istanbul, 34134, Turkiye
[5]Istanbul Health and Technology University, Vocational School of Health, Anesthesiology and Reanimation Department, Istanbul, 34275, Turkiye
[!]aynur.muduroglu@nisantasi.edu.tr
[*]Deceased

1 Supplementary Information

1.1 Imaginary Part of the Coherence Calculation

The coherency between channels i and j, denoted as $C_{ij}(f)$, was computed using the following formula:

$$C_{ij}(f) = \frac{S_{ij}(f)}{\sqrt{S_{ii}(f)S_{jj}(f)}} \tag{1}$$

Where $S_{ij}(f)$ represents the cross-spectrum between channels i and j, calculated as:

$$S_{ij}(f) = \langle x_i(f) x_j^*(f) \rangle \tag{2}$$

The iCOH values were calculated across specific frequency bands using:

$$iCOH(f) = \mathrm{Im}\left[C_{ij}(f)\right] \tag{3}$$

Here:

- $x_i(f)$ and $x_j(f)$: Fourier transform of the time series for channels i and j,
- *: Complex conjugation operation,
- $\langle\rangle$: Expectation values,
- $\mathrm{Im}[\cdot]$: Imaginary part of the coherency.

1.2 Global Efficiency Calculation

Global efficiency (GE) quantifies functional integration within a network. For a graph G with N nodes, the global efficiency is calculated using the formula:

$$GE = \frac{1}{N(N-1)} \sum_{i \neq j \in G} \frac{1}{d_{i,j}} \tag{4}$$

Here:

- N: Total number of nodes (or channels) in the graph,
- $d_{i,j}$: Shortest distance between nodes i and j,
- G: Graph representing the brain network.

1.3 Local Efficiency Calculation

The local efficiency of node i is calculated using the formula:

$$LE_i = \frac{1}{N-1} \sum_{j \neq i} \mathrm{GE}(G_i) \tag{5}$$

Here:

- N: Total number of nodes (or channels) in the graph,
- G_i: Subgraph consisting of the neighbors of node i,
- $\mathrm{GE}(G_i)$: Global efficiency of the subgraph G_i.

1.4 Transitivity Calculation

For a graph G with N nodes, transitivity is calculated using the formula:

$$T = \frac{\sum_{i \in N} 2t_i}{\sum_{i \in N} k_i(k_i - 1)} \tag{6}$$

Where:

- t_i: Geometric mean of the weights for all possible triangles around a given node i, calculated using Equation 8,
- k_i: Degree of a node i, computed using Equation 7,
- N: Total number of nodes in the graph.

The node degree k_i is calculated as:

$$k_i = \sum_{j=1}^{N} a_{ij} \tag{7}$$

Here, a_{ij} represents the number of edges between nodes i and j.
The geometric mean t_i of the weights w for all possible triangles around a given node i is given by:

$$t_i = \frac{1}{2} \sum_{j,h \in N} \sqrt[3]{w_{ij} w_{jh} w_{ih}} \tag{8}$$

Where:

- w_{ij}, w_{jh}, w_{ih}: Weights of the edges forming a triangle with node i,
- $j, h \in N$: Nodes forming a triangle with i.

1.5 Fractal Dimension Calculation

For a given time series x_i of length n, the Katz Fractal Dimension (FD) is calculated with the following formulas:

The total length of the waveform (L) is calculated using:

$$L = \sum_{i=1}^{n-1} \sqrt{(x_{i+1} - x_i)^2 + 1} \tag{9}$$

The diameter of the waveform (d) is given by:

$$d = \max\left(\sqrt{(x_i - x_1)^2 + (1 - i)^2}\right) \tag{10}$$

Using these values, the Katz fractal dimension (FD) is computed as:

$$FD = \frac{\log(n)}{\log(n) + \log(d/L)} \tag{11}$$

Here:

- n: Total number of data points in the time series,
- x_i: The i-th data point in the time series,
- L: Total waveform length of the time series,
- d: Maximum waveform diameter.

1.6 Quadratic Sample Entropy Calculation

SampEn is computed as:

$$\text{SampEn} = -\ln\left(\frac{A^m(r)}{B^m(r)}\right) \tag{12}$$

Here:

- m: Signal segment length,
- r: Tolerance,
- B_i: Count of matches at a distance of m for each segment,
- A_i: Count of matches at a distance of $m+1$.

To remove the dependency of SampEn on r, this probability function is normalized by dividing by twice the tolerance window, yielding the QSE criterion:

$$\text{QSE} = \text{SampEn} + \ln(2r) \tag{13}$$

In our case, QSE was computed for $r = 0.1, 0.2, 0.3, \ldots, 1.00$ and for $m = 1$ and $m = 2$. The parameters were selected to obtain the lowest p value in the Wilcoxon signed rank test.

1.7 Quantile Graph Calculation

Let $\mathscr{M}$ be a map from a time series $X \in T$ to a network $g \in G$ and time series partitioned into Q quantiles, denoted as $q_1, q_2, ..., q_Q$. Then:

$$\mathscr{M} \ : X, t \rightarrow g, q \tag{14}$$

$$g \ = \{N, A\} \tag{15}$$

Here:

- N: Nodes
- A: Edges

Then we create the adjacency matrix as follows for $t = 0, 1, 2, ..., T$:

$$A_{ij} = \begin{cases} +1, & \text{if } \mathscr{M}(x(t)) = q_i \,\&\, \mathscr{M}(x(t+k)) = q_j, \\ 0, & \text{otherwise.} \end{cases} \tag{16}$$

$$\mathscr{N}(A) = W_k \tag{17}$$

Here:

- A_{ij}: Adjacency Matrix
- W_k: Markov transition matrix

In this study, the average jump length is chosen for a topological feature. The average jump length ($\Delta(k)$) can be calculated with following Equation 18:

$$\Delta(k) = \frac{1}{Q} tr(PW_k^T) \tag{18}$$

Here:

- W_k^T: Transpose of the Markov transition matrix
- P: Distance between quantiles
- tr: Trace operator

According to the Freedman-Diaconis rule QG was computed with $N_{QG} = Q = 2(1,600)1/3 \approx 23$, using $k = 1, 2, 3, ..., 25$.

1.8 Visibility Graph Calculation

A point in the time series is taken as a node. The visibility between i and j nodes is checked with a trigonometric formula that controls the k points between i and j, seen in Equation 19:

$$x(k) \leqslant x(j) + (x(i) - x(j)) \frac{j-k}{j-i} \tag{19}$$

With this verification adjacency matrix $A_{T \times T}$ is created. Then Graph Index Complexity (GIC) is calculated as a VG feature following:

$$\text{GIC} = 4c(1-c) \tag{20}$$

Where c is computed with the formula in Equation 21.

$$c = \frac{\lambda_{\max} - 2\cos\left(\frac{\pi}{N+1}\right)}{N - 1 - 2\cos\left(\frac{\pi}{N+1}\right)} \tag{21}$$

Here:

- $\lambda_{\max}$: The largest eigenvalue of the adjacency matrix
- N: Number of nodes

Lanczos algorithm is employed to reduce the computational cost of eigenvalue calculation (m = 10). VG had $N_{VG} = T = 1,600$.

1.9 Correlation Analysis Between EEG Metrics and Relative Abundance of Microbiota Taxa

Table S1. Exploratory correlation analyses between EEG metrics (VG, QG, QSE, FD across frequency bands and electrode sites) and gut microbiota taxa across multiple taxonomic levels. Both Kendall's τ and Spearman's ρ are reported with their respective two-sided p-values. Multiple comparison correction was applied using the Benjamini–Hochberg method, yielding q_{FDR} values. The column n denotes the number of valid subject pairs included in each analysis. Due to the exploratory scope and small sample size for microbiota profiling, results should be considered preliminary and hypothesis-generating.

EEG_feat	Taxon	Rank	p	τ	q_{FDR}	(Spearman) p	ρ	n
VG_Beta3_Fz	Aminipila	Genus	0.0001	0.769	0.795	0.00001	0.912	13
QG_Theta_Fp2	Dysosmobacter welbionis	Species	0.0001	0.813	0.795	0.00000	0.933	13
VG_Beta3_Fz	Aminipila terrae	Species	0.0001	0.744	0.795	0.00003	0.901	13
QSE_Gamma_Pz	Dysosmobacter welbionis	Species	0.0002	0.787	0.795	0.00001	0.916	13
QG_Delta_C4	Faecalicatena	Genus	0.0002	-0.787	0.795	0.00003	-0.897	13
QG_Delta_C4	Faecalicatena sp. Marseille-Q4148	Species	0.0002	-0.787	0.795	0.00003	-0.897	13
QSE_all_T3	Ethanoligenens	Genus	0.0002	0.825	0.795	0.00001	0.914	13
QSE_all_T3	Ethanoligenens harbinense	Species	0.0002	0.825	0.795	0.00001	0.914	13
QSE_Beta3_Fz	Negativicutes	Class	0.0003	0.718	0.795	0.00015	0.863	13
FD_Beta3_Cz	Mediterraneibacter	Genus	0.0003	-0.718	0.795	0.00027	-0.846	13
QSE_Delta_Cz	Lactobacillaceae	Family	0.0003	0.761	0.795	0.00004	0.894	13
QSE_Gamma_C4	Dysosmobacter welbionis	Species	0.0003	0.761	0.795	0.00001	0.916	13
FD_all_T4	Coriobacteriales	Order	0.0003	-0.774	0.795	0.00006	-0.886	13
FD_all_T4	Coriobacteriaceae	Family	0.0003	-0.774	0.795	0.00006	-0.886	13
FD_all_T4	Collinsella	Genus	0.0003	-0.774	0.795	0.00006	-0.886	13
FD_all_T4	Collinsella aerofaciens	Species	0.0003	-0.774	0.795	0.00006	-0.886	13
VG_Delta_T6	Faecalibacterium	Genus	0.0005	0.745	0.795	0.00006	0.884	13
VG_Delta_T6	Faecalibacterium prausnitzii	Species	0.0005	0.745	0.795	0.00006	0.884	13
VG_Theta_T5	Acutalibacter	Genus	0.0005	-0.745	0.795	0.00015	-0.862	13
VG_Theta_T5	Acutalibacter muris	Species	0.0005	-0.745	0.795	0.00015	-0.862	13
FD_Beta1_T4	Coriobacteriales	Order	0.0005	-0.747	0.795	0.00031	-0.841	13
FD_Beta1_T4	Coriobacteriaceae	Family	0.0005	-0.747	0.795	0.00031	-0.841	13
FD_Beta1_T4	Collinsella	Genus	0.0005	-0.747	0.795	0.00031	-0.841	13
FD_Beta1_T4	Collinsella aerofaciens	Species	0.0005	-0.747	0.795	0.00031	-0.841	13
QG_Delta_T6	Faecalibacterium duncaniae	Species	0.0005	-0.747	0.795	0.00003	-0.897	13
QSE_Delta_T6	Faecalibacterium duncaniae	Species	0.0005	-0.747	0.795	0.00006	-0.886	13
QSE_Theta_F7	Solibaculum	Genus	0.0005	0.692	0.795	0.00027	0.846	13
QSE_Theta_F7	Solibaculum mannosilyticum	Species	0.0005	0.692	0.795	0.00027	0.846	13
VG_Gamma_Fz	Aminipila	Genus	0.0005	0.692	0.795	0.00022	0.852	13
FD_Beta2_Cz	Mediterraneibacter	Genus	0.0005	-0.692	0.795	0.00053	-0.824	13
QSE_Beta1_Pz	Blautia argi	Species	0.0006	-0.756	0.795	0.00019	-0.855	13
QG_Beta2_Fp2	Veillonellales	Order	0.0007	0.765	0.795	0.00016	0.860	13
QG_Beta2_Fp2	Veillonellaceae	Family	0.0007	0.765	0.795	0.00016	0.860	13
QSE_all_O1	Ethanoligenens	Genus	0.0007	0.765	0.795	0.00006	0.884	13
QSE_all_O1	Ethanoligenens harbinense	Species	0.0007	0.765	0.795	0.00006	0.884	13
QG_Beta2_O1	Ethanoligenens	Genus	0.0007	-0.765	0.795	0.00003	-0.896	13
QG_Beta2_O1	Ethanoligenens harbinense	Species	0.0007	-0.765	0.795	0.00003	-0.896	13
VG_Beta1_T4	Lachnoclostridium phocaeense	Species	0.0007	-0.765	0.795	0.00006	-0.884	13
FD_Delta_C3	Maliibacteriaceae	Family	0.0007	-0.741	0.795	0.00011	-0.870	13

EEG_feat	Taxon	Rank	p	τ	q_{FDR}	(Spearman) p	ρ	n
FD_Delta_C3	Maliibacterium	Genus	0.0007	-0.741	0.795	0.00011	-0.870	13
FD_Delta_C3	Maliibacterium massiliense	Species	0.0007	-0.741	0.795	0.00011	-0.870	13
VG_Delta_T4	Clostridium butyricum	Species	0.0007	0.719	0.795	0.00027	0.845	13
QG_Delta_T4	Clostridium butyricum	Species	0.0007	-0.719	0.795	0.00008	-0.878	13
QSE_Delta_Fz	Coprobacillaceae	Family	0.0007	-0.719	0.795	0.00012	-0.867	13
QSE_Delta_T3	Ruminococcus champanellensis	Species	0.0007	-0.719	0.795	0.00015	-0.862	13
QG_Gamma_C4	Turicibacteraceae	Family	0.0008	-0.882	0.795	0.00006	-0.937	10
QG_Gamma_C4	Turicibacter	Genus	0.0008	-0.882	0.795	0.00006	-0.937	10
QG_Theta_Oz	Dysosmobacter welbionis	Species	0.0008	0.710	0.795	0.00051	0.825	13
QSE_Beta3_F8	Dysosmobacter welbionis	Species	0.0008	0.710	0.795	0.00016	0.861	13
QG_Alpha_P4	Faecalicatena	Genus	0.0008	-0.710	0.795	0.00013	-0.867	13
QG_Alpha_P4	Faecalicatena sp. Marseille-Q4148	Species	0.0008	-0.710	0.795	0.00013	-0.867	13
QG_Theta_Oz	Lachnospira	Genus	0.0008	0.721	0.795	0.00014	0.864	13
QG_Theta_Oz	Lachnospira eligens	Species	0.0008	0.721	0.795	0.00014	0.864	13
VG_Delta_T6	Faecalibacterium duncaniae	Species	0.0008	0.721	0.795	0.00006	0.883	13
FD_Beta2_T4	Coriobacteriales	Order	0.0008	-0.721	0.795	0.00037	-0.836	13
FD_Beta2_T4	Coriobacteriaceae	Family	0.0008	-0.721	0.795	0.00037	-0.836	13
FD_Beta2_T4	Collinsella	Genus	0.0008	-0.721	0.795	0.00037	-0.836	13
FD_Beta2_T4	Collinsella aerofaciens	Species	0.0008	-0.721	0.795	0.00037	-0.836	13
FD_Beta3_Oz	Coriobacteriales	Order	0.0008	-0.721	0.795	0.00034	-0.839	13
FD_Beta3_Oz	Coriobacteriaceae	Family	0.0008	-0.721	0.795	0.00034	-0.839	13
FD_Beta3_Oz	Collinsella	Genus	0.0008	-0.721	0.795	0.00034	-0.839	13
FD_Beta3_Oz	Collinsella aerofaciens	Species	0.0008	-0.721	0.795	0.00034	-0.839	13
FD_Beta3_T4	Coriobacteriales	Order	0.0008	-0.721	0.795	0.00018	-0.858	13
FD_Beta3_T4	Coriobacteriaceae	Family	0.0008	-0.721	0.795	0.00018	-0.858	13
FD_Beta3_T4	Collinsella	Genus	0.0008	-0.721	0.795	0.00018	-0.858	13
FD_Beta3_T4	Collinsella aerofaciens	Species	0.0008	-0.721	0.795	0.00018	-0.858	13
FD_Gamma_T4	Coriobacteriales	Order	0.0008	-0.721	0.795	0.00018	-0.858	13
FD_Gamma_T4	Coriobacteriaceae	Family	0.0008	-0.721	0.795	0.00018	-0.858	13
FD_Gamma_T4	Collinsella	Genus	0.0008	-0.721	0.795	0.00018	-0.858	13
FD_Gamma_T4	Collinsella aerofaciens	Species	0.0008	-0.721	0.795	0.00018	-0.858	13
QG_all_Pz	Blautia	Genus	0.0009	-0.833	0.795	0.00094	-0.900	9
QG_Gamma_C3	Alistipes onderdonkii	Species	0.0009	-0.776	0.795	0.00018	-0.877	12
FD_all_F3	Enterocloster clostridioformis	Species	0.0009	0.667	0.795	0.00053	0.824	13
QG_Theta_Oz	Oscillospiraceae	Family	0.0009	0.667	0.795	0.00032	0.841	13
QSE_all_F7	Enterocloster clostridioformis	Species	0.0009	0.667	0.795	0.00097	0.802	13
VG_Alpha_O1	Eubacteriales Family XIII. Incertae Sedis	Family	0.0009	0.667	0.795	0.00128	0.791	13
VG_Gamma_F8	Aminipila terrae	Species	0.0009	0.667	0.795	0.00038	0.835	13
VG_Gamma_T5	Aminipila	Genus	0.0009	0.667	0.795	0.00045	0.830	13
VG_Gamma_T6	Aminipila terrae	Species	0.0009	0.667	0.795	0.00084	0.808	13
VG_Theta_F4	Lacrimispora saccharolytica	Species	0.0009	0.667	0.795	0.00097	0.802	13
QG_Alpha_Fpz	(unclassified Lachnospiraceae)	Genus	0.0009	-0.667	0.795	0.00111	-0.797	13
QG_Delta_Fp2	Eubacteriales Family XIII. Incertae Sedis	Family	0.0009	-0.667	0.795	0.00015	-0.863	13
QSE_Delta_P3	Bacteroidota	Phylum	0.0009	-0.667	0.795	0.00145	-0.786	13
QSE_Delta_P3	Bacteroidia	Class	0.0009	-0.667	0.795	0.00145	-0.786	13
QSE_Delta_P3	Bacteroidales	Order	0.0009	-0.667	0.795	0.00145	-0.786	13
QG_Gamma_T4	Bacilli	Class	0.0009	-0.778	0.795	0.00117	-0.867	10
QG_Gamma_T4	Lactobacillales	Order	0.0009	-0.778	0.795	0.00117	-0.867	10
QG_Beta3_Cz	Ruthenibacterium	Genus	0.0010	0.697	0.795	0.00026	0.867	12
QG_Beta3_Cz	Ruthenibacterium lactatiformans	Species	0.0010	0.697	0.795	0.00026	0.867	12
